# Relations between speech‐reception, psychophysical temporal processing, and subcortical electrophysiological measures of auditory function in humans

**DOI:** 10.1016/j.heares.2022.108456

**Published:** 2022-04

**Authors:** Samuele Carcagno, Christopher J. Plack

**Affiliations:** a Department of Psychology, Lancaster University, Lancaster LA1 4YF, United Kingdom; b Manchester Centre for Audiology and Deafness, University of Manchester, Manchester Academic Health Science Centre, Manchester M13 9PL, United Kingdom; c Brain and Cognition Research Centre, University of Toulouse Paul Sabatier, Toulouse, France; d Brain and Cognition Research Centre, CNRS, UMR-5549, Toulouse, France

**Keywords:** Cochlear synaptopathy, Presbycusis, Temporal coding, Hearing loss, Speech reception, Pitch, ABR, Auditory brainstem response, ABRQ, Auditory brainstem response in quiet, AMD, Amplitude modulation detection, AP, Action potential, C7, Seventh cervical vertebra, CNT, Centered, COG, Cognitive, cons, Consonance, CRM, Coordinate response measure, CS, Cochlear synaptopathy, diff, difference, DTT, Digit triplets test, elec, electrophysiological, ENV, Envelope, ERL, Earlobe, F0D, F0 discrimination, FD, Frequency discrimination, FFR, Frequency following response, HF, High forehead, HL, High level, HP, Highpass, IHC, Inner hair cell, IPD, Interaural phase difference, L/M-SR, Low and medium spontaneous rate, LERL, Linked earlobes, LL, Low level, LMST, Linked mastoids, LTPR, Linked tiptrodes, MI, Modulation index, MLR, Multiple linear regression, MOD, Modulator, OFF, Offset, PC, Principal component, PCA, Principal component analysis, psyphy, psychophysical, PT, Pure tone, PTA, Pure tone average (audiometric thresholds), PV, Proportion of variance, SLR, Simple linear regression, SNR, Signal-to-noise ratio, SP, Summating potential, TCNE, Total cumulative noise exposure, TFS, Temporal fine structure, TPR, Tiptrode, UML, Updated maximum likelihood, UPV, Unique proportion of variance

## Abstract

There is a large amount of variability in performance in masked-speech reception tasks, as well as in psychophysical auditory temporal processing tasks, between listeners with normal or relatively normal low-frequency hearing. In this study we used a cross-sectional dataset collected on 102 listeners (34 young, 34 middle-aged, 34 older) to assess whether variance in these tasks could be explained by variance in subcortical electrophysiological measures of auditory function (auditory brainstem responses and frequency following responses), and whether variance in speech-reception performance could be explained by variance in auditory temporal processing tasks. The potential confounding effect of high-frequency sensitivity was strictly controlled for by using highpass masking noise. Because each high-level construct (masked-speech reception, auditory temporal processing, and subcortical electrophysiological function) was indexed by several variables, we used principal component analyses to reduce the dimensionality of the dataset. Multiple-regression models were then used to assess the associations between the extracted principal components while controlling for a range of possible confounders including age and audiometric thresholds. We found that masked-speech reception was credibly associated with psychophysical auditory temporal processing abilities. No credible associations were found between masked-speech reception and electrophysiological measures of subcortical auditory function, or between psychophysical measures of auditory temporal processing and electrophysiological measures of subcortical auditory function. These results suggest that either the electrophysiological measures of subcortical auditory function used were not sufficiently sensitive to the subcortical neural processes limiting performance in the speech-reception and psychophysical auditory temporal-processing tasks, or that variance in these tasks is largely unrelated to variance in subcortical neural processes in listeners with near-normal hearing.

## Highlights

• Masked-speech reception is associated with auditory temporal-processing performance.
• No evidence that subcortical EEG measures are related to speech-reception performance.
• No evidence that subcortical EEG measures are related to auditory temporal processing.

## 1 Introduction

There is a large amount of inter-individual variability in auditory abilities, such as the ability to understand masked speech, or the ability to discriminate sounds on the basis of auditory attributes, such as pitch or amplitude modulation (Kidd, Watson, Gygi, 2007, Oxenham, 2016). Although losses of hearing sensitivity in some individuals can sometimes partly account for this variance, usually large proportions of variance remain unaccounted for (Humes, 2013, Humes, Dubno, 2010, Moore, 2007).

Variability in subcortical auditory processing has been hypothesized to be an important factor in explaining variability in auditory abilities (Felix et al., 2018). Innate differences, as well as environmental factors such as noise exposure (Kujawa and Liberman, 2009), aging (Sergeyenko, Lall, Liberman, Kujawa, 2013, Syka, 2020), or disease (AlJasser et al., 2020) could affect the integrity and functioning of the auditory nerve and auditory brainstem nuclei and drive the variance observed in auditory abilities. Subcortical temporal coding can be defined generally as the subcortical neural processing allowing a representation of the temporal features of a sound waveform, including its rapidly varying temporal fine structure (TFS) and its slowly varying temporal envelope (ENV) (Bharadwaj, Verhulst, Shaheen, Liberman, Shinn-Cunningham, 2014, Eggermont, 2015). It should be pointed out that temporal coding fidelity can be affected not only by the precision with which single neurons can phase lock to the temporal features of a sound waveform, but also, at the population level, by neural loss or by deafferentiation (Bharadwaj, Verhulst, Shaheen, Liberman, Shinn-Cunningham, 2014, Lopez-Poveda, 2014). Differences in subcortical temporal coding fidelity, which is thought to be important for the representation of several features of speech and musical signals (Moore, 2008, Moore, 2019, Picton, 2013, Plack, Barker, Prendergast, 2014), have been hypothesized to account for interindividual differences in both psychophysical tests of auditory temporal processing and tests of masked-speech reception (Bharadwaj, Verhulst, Shaheen, Liberman, Shinn-Cunningham, 2014, Plack, Barker, Prendergast, 2014), as well in the appreciation of musical attributes such as consonance (Bidelman, Krishnan, 2009, Bidelman, Krishnan, 2011, Bones, Hopkins, Krishnan, Plack, 2014). Additionally, the fact that both subcortical temporal coding fidelity and several auditory abilities tend to decline with age, has led to the hypothesis that age-related declines in these auditory abilities are driven by age-related declines in subcortical temporal processing (Eggermont, 2015, Felix, Gourévitch, Portfors, 2018).

The study of the relations between subcortical auditory function and auditory abilities in humans is limited by the fact that invasive recordings of subcortical structures cannot generally be performed, and by the fact that subcortical auditory processing cannot be directly manipulated. However, the study of the associations between scalp-recorded electrophysiological responses that are generated in subcortical structures, such as the auditory brainstem response (ABR) and the frequency following response (FFR), with auditory abilities offers a window to assess relations between subcortical auditory function and auditory abilities in humans. Both ABR wave amplitudes and FFR phase-locking strength can be affected by either neural deafferentiation/survival, or by the precision of phase-locked responses at the level of single neurons. ABR wave amplitudes can be reduced by jitter in the timing of neural responses because they depend on synchronous firing across large numbers of neurons (Bourien et al., 2014). A decrease in the number of fibers contributing to the response, due to deafferentiation, can also decrease ABR wave amplitudes (Kujawa and Liberman, 2009). The strength of phase locking to features of a sound waveform indexed by the FFR can be negatively affected not only by loss of phase-locking precision at the level of single neurons (Mamo et al., 2016), but also by reductions in the number of fibers contributing to the response (Encina-Llamas, Harte, Dau, Shinn-Cunningham, Epp, 2019, Shaheen, Valero, Liberman, 2015). ABR and FFR responses thus provide a precious window to assess subcortical neural auditory function in humans, which can then be related to auditory abilities.

Several studies on listeners with normal or near-normal hearing have confirmed an association between masked-speech reception and psychophysical measures of temporal processing such as frequency modulation, or interaural phase difference detection (Füllgrabe, Moore, Stone, 2014, Oberfeld, Klöckner-Nowotny, 2016, Schoof, Rosen, 2014). However, the results of studies investigating the relations between speech reception and electrophysiological measures of subcortical auditory function, or the relations between psychophysical measures of auditory temporal processing and electrophysiological measures of subcortical auditory function, have been mixed. A number of studies have reported significant associations between speech reception and electrophysiological subcortical auditory function measures (Liberman, Epstein, Cleveland, Wang, Maison, 2016, Mepani, Kirk, Hancock, Bennett, de Gruttola, Liberman, Maison, 2020, Valderrama, Beach, Yeend, Sharma, Van Dun, Dillon, 2018), while other studies have failed to find evidence for such associations (Bramhall, Konrad-Martin, McMillan, 2018, Guest, Munro, Prendergast, Millman, Plack, 2018, Johannesen, Buzo, Lopez-Poveda, 2019, Prendergast, Couth, Millman, Guest, Kluk, Munro, Plack, 2019, Prendergast, Millman, Guest, Munro, Kluk, Dewey, Hall, Heinz, Plack, 2017, Schoof, Rosen, 2016, Smith, Krizman, Liu, White-Schwoch, Nicol, Kraus, 2019). Similarly, associations between psychophysical measures of auditory temporal processing and electrophysiological measures of subcortical auditory function have been found by some studies (Bharadwaj, Masud, Mehraei, Verhulst, Shinn-Cunningham, 2015, Verhulst, Ernst, Garrett, Vasilkov, 2018), but not others (Paul, Waheed, Bruce, Roberts, 2017, Prendergast, Millman, Guest, Munro, Kluk, Dewey, Hall, Heinz, Plack, 2017).

In order to elucidate the relations between these measures, in the current study we exploited a rich dataset that we previously analyzed with the aim of finding signs of age-related cochlear synaptopathy (CS), or more generally, of age-related declines that could not be accounted for by loss of hearing sensitivity (Carcagno, Plack, 2020, Carcagno, Plack, 2021). This dataset includes tests of abilities close to real-world hearing abilities (speech reception, and assessment of preference for musical consonance, which for brevity will be referred to as tests of real-world hearing abilities), psychophysical tests of auditory temporal processing, and electrophysiological tests of subcortical auditory processing collected on a cross-sectional sample of 102 participants ranging in age from 19 to 74 years. In the current study, we used this dataset to answer the following questions: i) to what extent can measures of real-world hearing abilities be predicted by electrophysiological measures of subcortical auditory function? ii) to what extent can measures of real-world hearing abilities be predicted by psychophysical measures of auditory temporal processing? and, iii) to what extent can psychophysical measures of auditory temporal processing be predicted by electrophysiological measures of subcortical auditory function? These questions are important because associations between the three constructs mentioned above can provide insights into the mechanisms underlying individual differences in auditory abilities.

The dataset we used in the current study is ideally suited to address the above questions not only because it contains measurements for each of the three constructs (real-world hearing abilities, psychophysical temporal processing, and subcortical auditory function) collected from a large sample of participants, but also because each construct was assessed using several different tests, at different stimulus levels and in some cases different stimulus frequencies, thus providing a comprehensive assessment of each construct. Given the large number of different measures used to assess each construct, in order to improve the interpretability of the results we used principal component analysis (PCA; Jolliffe, 2002, Sharma, 1996) to reduce the number of variables while preserving the largest sources of variance in the measures. The dataset also contains measurements of audiometric thresholds, cognitive abilities, and noise exposure, thus allowing us to control statistically for the potential confounding effects that these variables may have on the associations between the different constructs. Moreover, we took two additional measures in the collection of the dataset to minimize the potentially confounding effect of high-frequency hearing sensitivity on behavioral and electrophysiological measures. These measures included selecting listeners with relatively preserved hearing sensitivity within a low-frequency region below ∼ 4 kHz, and presenting the stimuli within this low-frequency region in the presence of high-pass masking noise to eliminate the contribution of higher cochlear frequency regions to the electrophysiological or behavioral responses.

Although the main analyses addressed questions i and iii listed above using electrophysiological measures obtained in high-pass masking noise in order to match the frequency regions contributing to the behavioral and electrophysiological responses, we performed additional exploratory analyses addressing the same questions, but using ABR responses recorded in quiet. Although such responses reflect the contribution of high-frequency regions that did not contribute directly to performance in the behavioral tests, it is possible that the physiological status of these regions is indirectly associated with physiological dysfunctions in low-frequency cochlear regions that may not be captured by the frequency-specific electrophysiological responses. The additional exploratory analyses using the ABR in quiet aimed to assess this possibility. Finally, additional exploratory analyses addressing questions i–iii were performed using differential measures contrasting responses obtained at low and at high stimulus levels. A possible cause of interindividual variability in auditory temporal processing is CS, which is thought to mainly affect responses at high stimulus levels (Bharadwaj, Verhulst, Shaheen, Liberman, Shinn-Cunningham, 2014, Plack, Leger, Prendergast, Kluk, Guest, Munro, 2016). If this is the case, and if CS does indeed make major contributions to the variance in auditory temporal processing, then differential measures contrasting responses at high and low stimulus levels (Plack et al., 2016) could be more sensitive in the assessment of auditory temporal processing than simple measures because they would minimize between-subject variance due to nuisance factors (e.g. variability in electrophysiological responses due to interindividual differences in head size or myogenic activity). The additional exploratory analyses using the differential measures were thus run to address the study questions using these potentially more sensitive measures.

## 2 Methods

### 2.1 Participants

A total of 170 participants (129 females) from three age groups (young: 18–39, middle-aged: 40–59, older: >60 years old) were enrolled in the study. Sixty-eight participants either failed to meet the selection criteria outlined below, or withdrew from the study. Only the data of the 102 participants who completed the study will be presented. Selection criteria included audiometric thresholds for both ears below 20 dB HL at octave frequencies from 0.125 to 2 kHz, and below 40 dB HL at 4 kHz. No selection criteria were imposed for frequencies above 4 kHz. Due to the use of an incorrect calibration table for the headphones used in the audiometric tests the actual cutoff thresholds differed by a few dBs with respect to the nominal cutoff thresholds listed above. Using the correct calibration table five older, two middle-aged, and two young participants would not have passed the selection. However, these listeners had thresholds below 30.5 dB HL for audiometric frequencies up to 2 kHz, and below 37 dB HL at 4 kHz. Given that their thresholds were only slightly above the cutoff criteria, and given that audiometric thresholds were used as continuous covariates, the data of these listeners were included in the analyses. Participants with audiometric threshold asymmetries between the left and right ear larger than 20 dB at any frequency from 0.125 to 4 kHz were excluded from the study. An otoscopic examination was performed prior to the beginning of the tests, and participants with earwax occlusions were excluded from the study. Participants were required to be native British English speakers.

Recruitment continued until 34 participants from each age group had completed the study. The youngest participant was 18.8, while the oldest was 73.6 years old. Within each age group 27 females, and seven males completed the study. Towards the end of the study recruitment was targeted to ensure that the proportion of females to males would be the same across the three age groups. The larger number of females present in the final sample of participants largely reflects the fact that a larger number of females enrolled in the study, but partly reflects the fact that a higher proportion of males (22%; one middle-aged, eight older) than females (9%; two young, three middle-aged, seven older) who enrolled in the study failed to meet our audiometric inclusion criteria. The fact that a larger proportion of females enrolled in the study may also be partly related to better hearing thresholds for females than males in the general population, because the adverts for the study called for participants who had not been diagnosed with a low-frequency hearing loss. The higher proportion of older males with hearing loss observed in our sample is consistent with the results of epidemiological studies (Dubno, Eckert, Lee, Matthews, Schmiedt, 2013, Fitzgibbons, Gordon-Salant, 2010). These sex differences in hearing sensitivity are thought to be largely due to lifestyle differences (e.g. occupational noise exposure) leading to greater noise exposure in males, and appear to be decreasing in studies conducted in recent years (Homans et al., 2017), possibly as a result of better protection from occupational noise exposure in modern societies. There are also known sex differences in ABR wave amplitudes (Don et al., 1993). Although the greater proportion of females than males in our sample may potentially limit the generalizability of our findings, we are not aware of any evidence suggesting that sex differences would bias the relations between the measures reported in the current study. For example, while the higher ABR wave amplitudes generally observed in females may make it easier to record ABRs to low-level stimuli close to the noise floor, and thus reduce the standard errors of correlations between ABR wave amplitudes and masked-speech reception thresholds, or with thresholds in psychophysical temporal processing tasks, we are not aware of evidence that this would bias the direction of such correlations.

Participants were asked to report the number of years of musical practice (with a musical instrument or vocal) they had. They gave written informed consent for participation in the study, and received an hourly wage. All the experimental procedures were approved by the Lancaster University Research Ethics Committee.

### 2.2 Test battery

The test battery has been described in detail in previous publications (Carcagno, Plack, 2020, Carcagno, Plack, 2021), therefore, only a high-level overview of the measures will be given here. Most tasks were run in several conditions. All the psychophysical, speech-reception, consonance preference, and ABR measures were obtained at a lower [thought to engage minimally auditory nerve fibers with low and medium spontaneous firing rates (L/M-SR fibers)] and at a higher stimulus level (thought to engage maximally L/M-SR fibers; Bharadwaj, Verhulst, Shaheen, Liberman, Shinn-Cunningham, 2014, Plack, Barker, Prendergast, 2014). The rationale for this choice is linked to our two previous studies, which contrasted responses at high and low stimulus levels to obtain putative measures of CS. According to major models of this syndrome these measures should be more sensitive, and more specific, than non-differential measures (Bharadwaj, Verhulst, Shaheen, Liberman, Shinn-Cunningham, 2014, Plack, Leger, Prendergast, Kluk, Guest, Munro, 2016). However, the use of different stimulus levels is beneficial also for the current study, as it allows a more comprehensive assessment of auditory function at different operating points compared to the use of a single stimulus level. For the psychophysical, speech-reception, and consonance preference tasks, the low- and high-level stimulus root mean square levels were close to 40 and 80 dB SPL respectively (see Carcagno and Plack, 2021, for details). The ABRs were obtained with clicks at levels of 80 and 105 dB ppeSPL. The FFR ENV measures were obtained for stimuli presented all at a high level (75 dB SPL carriers), but amplitude modulated with a lower or higher modulation index (MI), which should also engage differentially L/M-SR fibers (with higher engagement for high-level stimuli with a lower MI according to auditory modeling simulations; Bharadwaj et al., 2014). For all the psychophysical, speech-reception, and consonance preference tasks the high-pass masking noise consisted of a pink noise bandpass filtered between 3 and 8 kHz, with a spectrum level at 4 kHz that was ∼ 40 dB below the overall level of the stimuli. For the electrophysiological recordings the high-pass masking noise consisted of a pink noise that was bandpass filtered between 3 and 8 kHz for the FFR, and between 3.5 and 8 kHz for the ABR recordings. The spectrum level of this noise for the FFR recordings was 50 dB SPL at 4 kHz, while for the ABR it had a spectrum level of 40 and 65 dB SPL at 1 kHz, respectively for the 80, and 105 dB ppeSPL clicks. During pilot studies the high-pass masking noise levels chosen for the electrophysiological responses were found to completely mask the electrophysiological responses when the noises, instead of being high-pass filtered, were presented also within the frequency region of the stimuli.

Besides level or MI differences, for most tasks there were also additional conditions determined by e.g. the stimulus frequency, modulation rate or other factors. For tasks in which the stimulus frequency was varied, frequencies around 0.6 and 2 kHz were used to probe for effects at different cochlear frequency regions that still lay within the larger low-frequency region (below ∼ 3 kHz) in which thresholds were near-normal for all participants. For modulated stimuli, a modulation rate ∼ 100 Hz was chosen as a compromise to ensure that side-bands would not be resolved for 2-kHz carriers (Glasberg and Moore, 1990) while the FFR would reflect mainly subcortical sources (Bharadwaj, Mai, Simpson, Choi, Heinz, Shinn-Cunningham, 2019, Bidelman, 2018, Bidelman, Momtaz, 2021). Additional lower modulation rates were used for the amplitude modulation detection (AMD) task to allow for a more fine-grained assessment of age effects at different modulation frequencies. For brevity, variables will be abbreviated with a prefix consisting of the task name (e.g. FD for frequency discrimination), followed (where appropriate) by an indication of the stimulus level (LL for low level, HL for high level) or the MI, and other suffixes needed to distinguish any additional factors; for example, pure tone frequency discrimination at the low stimulus level at 2 kHz will be abbreviated as FD_LL_2kHz.

The stimuli for the electrophysiological measures were presented via insert earphones (ER-3A; Etymotic Research Inc., Elk Grove, U.S.A.), while the stimuli for the psychophysical temporal processing tasks and for the tests of real-world hearing abilities were presented via circumaural headphones (HD650; Sennheiser electronic GmbH & Co. KG, Hanover, Germany).

Psychophysical and speech reception tests were run with *n*-interval *m*-alternative forced-choice tasks using the updated maximum likelihood (UML) adaptive procedure (Shen and Richards, 2012). Thresholds, estimated by fitting psychometric functions, were used as a measure of performance.

#### 2.2.1 Speech reception measures

Speech reception was assessed with the coordinate response measure (CRM; Bolia et al., 2000), and the digit triplets test (DTT; Smits et al., 2004). For the CRM, thresholds were measured both with speech maskers colocated (CNT) with the target speech (that was always presented at a 0${}^{\circ }$ azimuth), or offset (OFF) by a $\pm 65^{\circ }$ azimuth. Spatialization was achieved by convolving the sentences with the head-related impulse responses of subject #3 from the CIPIC database (Algazi et al., 2002). The DTT test was run with a noise lowpass filtered at 3 kHz. The noise was intended to be a speech-shaped noise, but due to a bug in the software used to generate it, it had a slightly different spectral shape (see Carcagno and Plack, 2021).

#### 2.2.2 Consonance preference

Preference for consonant musical intervals was assessed by subtracting ratings for dissonant dyads (tritones) from ratings for consonant dyads (perfect fifths). Although ratings were collected for different fundamental frequencies (F0s) this was not a factor of interest in the current study, and the consonance (cons) task resulted in only two variables: consonance preference at a low stimulus level (cons_LL), and consonance preference at a high stimulus level (cons_HL).

#### 2.2.3 Psychophysical temporal processing measures

Psychophysical temporal processing measures comprised thresholds for the detection of sinusoidal amplitude modulation with pure tone carriers, frequency/F0 discrimination (FD and F0D tasks), and interaural phase difference detection (IPD task). The AMD task was run at three modulation rates (25, 50, and 100 Hz). The FD task was run with 0.6 and 2 kHz pure tones. The F0D task was run with an unresolved complex tone with a 100-Hz F0, bandpass filtered between 1.5 and 2.5 kHz. The IPD task was run by introducing an interaural phase difference to the modulator (MOD condition) of an amplitude modulated tone, or for a 0.6 kHz pure (PT condition). For the MOD condition the carrier frequency was either 0.6 kHz, or 2 kHz.

#### 2.2.4 Auditory brainstem response

Click-evoked ABRs were recorded with or without highpass masking noise to eliminate the contribution of high-frequency cochlear regions, typically showing outer hair cell dysfunction in older people, to the response. Because most of the measures of this study were obtained in a low-frequency cochlear region that was relatively spared by outer hair cell dysfunction in the older participants (as indexed by audiometric thresholds), only the highpass masked ABRs were included in the main analyses. The ABRs obtained in quiet were only used for secondary exploratory analyses.

ABRs yielded four measures, wave I and V peak-trough amplitudes and latencies, for two electrode montages [high forehead (HF) referenced to the ipsilateral earlobe (ERL), and HF referenced to the ipsilateral tiptrode (TPR)]. PCA requires either data without missing values or the imputation of missing values. Both ABR amplitudes and latencies had some missing values due to peaks or troughs buried within the noise floor. For wave amplitudes the cause of missing values could be reasonably imputed to a very low underlying wave amplitude, and missing amplitude values were imputed as the lowest recorded amplitude value in the dataset (0.38 nV), which is practically close to zero. For wave latencies the reason for missing data was the same (low wave amplitude value), but an estimate of the underlying latency value cannot be inferred from this information. Improper imputation or an analysis limited to the cases with complete data could lead to biased results (Gelman and Hill, 2007). For this reason, the ABR wave latencies were not included in the current analyses.

#### 2.2.5 Frequency following response

FFRs were obtained for 0.6 and 2 kHz carriers modulated with a MI of 0.7 or 1 using four different montages: HF referenced to the 7th cervical vertebra (C7 montage), HF referenced to the linked earlobes (LERL montage), HF referenced to the linked mastoids (LMST montage), and HF referenced to the linked tiptrodes (LTPR montage). The addition of FFRs recorded in opposite polarities was used to derive ENV responses, while their subtraction was used to derive TFS responses. TFS responses were measured only for the 0.6 kHz carrier because the frequency of the 2 kHz carrier was too high to generate such responses (Krishnan, 2007). Furthermore, under the assumption that TFS responses should not be greatly affected by the MI, such responses were obtained by averaging across MIs. Both FFR amplitudes (estimated in the frequency domain via fast Fourier transforms and summarized by signal to noise ratios) and latencies (estimated via group delay) were obtained. FFR latencies, however, had missing values that may have not been missing at random or completely at random, hence could not be imputed in a straightforward way. For this reason, only FFR amplitudes were used for the analyses of the current paper. Overall there were 16 variables indexing FFR ENV amplitudes (given by the combination of two carriers, two MIs, and four electrode montages), and four variables indexing FFR TFS amplitudes (one for each electrode montage).

#### 2.2.6 Covariates

Covariates included audiometric thresholds, estimates of lifetime noise exposure, cognitive test scores, and years of musical experience (practice with musical instruments or singing). For the main analyses audiometric thresholds were summarized as the pure tone average at octave frequencies between 0.125 and 2 kHz (PTA_0.125-2_), a relatively narrow frequency region that covers the frequencies of the stimuli used in the other tests included in the analyses.

Estimates of lifetime noise exposure were obtained via the structured interview developed by Lutman et al. (2008) and were summarized as the log_10_ total (including recreational and occupational) cumulative noise exposure energy (log_10_TCNE), so that a unit difference in this measure corresponds to a tenfold difference in noise exposure energy.

Cognitive abilities were assessed with four tests (forward and backward digit span, reading span, and Raven’s progressive matrices). Scores on these tests were reduced via PCA resulting in one component accounting for 50% of the variance (COG_PC1; see supplementary materials for the PCA of cognitive scores).

Years of musical experience were estimated via participants’ self reports. Because the distribution of the number of years of musical experience was right skewed, a cube root transformation was applied to this variable before statistical analyses; this transformed variable will hereafter be referred to as MUS.

### 2.3 Principal component analyses

PCAs were computed in R (R Core Team, 2021) with the FactoMineR package (Lê et al., 2008). Variables were standardized before being entered into the PCAs. To address the main research questions presented in the Introduction four PCAs were run: i) one on the six variables indexing speech reception; ii) one on the two variables indexing consonance preference; iii) one on the 18 variables indexing psychophysical auditory temporal processing; iv) one on the 28 variables indexing the electrophysiological measures (ABR and FFR amplitudes). The number of PCs to retain was determined via parallel analysis perfomed with the psych R package (Revelle, 2019). Parallel analysis compares the eigenvalues of the PCA performed on a given dataset with those obtained from PCAs performed on random uncorrelated datasets of the same dimension and with the same sample size. Components with eigenvalues larger than the average eigenvalues obtained from the PCAs on random datasets are retained, while the remaining components likely reflect random error variability (Hayton et al., 2004).

### 2.4 Statistical analyses

The associations between the dependent and the independent variables were assessed via Bayesian robust multiple linear regression (MLR) models run in R (R Core Team, 2021) and JAGS (Plummer, 2003). Robust regression uses a Student’s *t* distribution instead of a Normal distribution for describing residuals, minimizing the potential influence of outliers on the estimated regression coefficients (Kruschke, 2014). Shrinkage priors were used for the slope coefficients in the models. These priors were described by a *t* distribution centered at zero, with 1 degree of freedom, and scale parameter set to 0.1. This prior assumes that the standardized slope coefficients should be generally close to zero, where the narrow peak of the *t* distribution is located, reflecting a belief that effect sizes will be generally small. However, owing to its heavy tails the *t* prior can accommodate coefficients much larger than zero if the likelihood provides clear evidence for this (Kruschke, 2014).

Standardized regression coefficients are reported as measures of effect size. The unique proportions of variance (UPV) explained by each independent variable of interest is also reported to gain further insight on the unique contribution that the variable makes to the model. The UPV is the additional variance explained by entering the independent variable of interest after entering all other variables. Additionally, the proportions of variance (PV) explained in the dependent variable in simple linear regressions (SLRs) with each independent variable of interest are reported. The difference in the SLR PV explained and the MLR UPV explained gives a measure of the effect of adding the covariates to the model. It should be noted that while the SLR PV explained will often be larger than the MLR UPV explained, this is not always the case: an independent variable can sometimes increase the overall PV explained by an MLR model by suppressing error variance in the covariates (Kim, 2019); in such cases the MLR UPV explained can be larger than the SLR PV explained.

## 3 Results

### 3.1 Principal component analyses

The eigenvalue and the percentage of variance explained by each PC extracted by the PCAs are shown in the supplementary materials (SM) for each PCA (Tables S1, S2, S3, S4). Parallel analysis indicated the extraction of one PC for the speech reception measures (speech_PC1, accounting for 48% of the variance), one PC for the two variables indexing consonance preference (cons_PC1, accounting for 85% of the variance), two PCs for the psychophysical measures of auditory temporal processing (psyphy_PC1 and psyphy_PC2, accounting together for 51% of the variance), and four PCs for the variables indexing the electrophysiological measures (elec_PC1, elec_PC2, elec_PC3, and elec_PC4, accounting together for 70% of the variance).

Inspection of the pattern of loadings shown in Table 1, indicates that speech_PC1 had positive loadings on all speech tests (high for the CRM tests and moderate for the DTT tests). cons_PC1 had high loadings, of 0.92, on the consonance preference test at both stimulus levels (the second PC, which was not included in further analyses had loadings of 0.38 for the low stimulus level condition and of -0.38 for the high stimulus level condition).

**Table 1:** Loadings (correlations) between PCs and variables of the PCA of speech tests scores. For brevity only the results for the first five PCs are shown.

	PC1	PC2	PC3	PC4	PC5
CRM_HL_CNT	0.64	-0.19	0.69	-0.00	-0.27
CRM_HL_OFF	0.83	-0.16	-0.29	-0.23	-0.27
CRM_LL_CNT	0.75	-0.40	0.14	0.20	0.46
CRM_LL_OFF	0.84	-0.14	-0.41	-0.08	0.02
DTT_HL	0.47	0.72	0.18	-0.42	0.22
DTT_LL	0.55	0.60	-0.09	0.56	-0.11

The patterns of loadings for the psychophysical temporal processing PCs are shown in Table 2. psyphy_PC1 accounted for 39% of the variance, and had positive loadings on all psychophysical temporal processing tests. The loadings were high for AMD tests, and moderate/high for IPD, FD, and F0D tests (except for F0D_LL, which was loaded weakly by this component). The pattern of loadings for psyphy_PC2, which accounted for 12% of the variance, was more complex: it had moderate positive loadings on the IPD tasks, low/moderate negative loadings on the AMD tasks, and close to zero loadings on the FD/F0D tasks. This component thus seems to reflect variance differentiating the IPD from the AMD tasks.

**Table 2:** Loadings (correlations) between PCs and variables of the PCA of psychophysical temporal processing tests scores. For brevity only the results for the first five PCs are shown.

	PC1	PC2	PC3	PC4	PC5
AM_HL_100Hz	0.81	-0.30	0.01	0.04	-0.09
AM_HL_25Hz	0.65	-0.52	-0.08	0.18	-0.05
AM_HL_50Hz	0.76	-0.47	-0.12	0.06	-0.07
AM_LL_100Hz	0.77	-0.23	-0.16	-0.17	0.16
AM_LL_25Hz	0.66	-0.41	-0.29	-0.07	0.03
AM_LL_50Hz	0.72	-0.29	-0.38	-0.12	0.16
F0D_HL	0.54	-0.16	0.49	0.25	-0.27
FD_HL_PT_2kHz	0.61	0.05	0.36	-0.17	0.40
FD_HL_PT_0.6kHz	0.57	0.16	0.53	-0.06	-0.13
F0D_LL	0.22	-0.09	0.48	0.43	0.16
FD_LL_PT_2kHz	0.49	0.18	0.28	-0.10	0.65
FD_LL_PT_0.6kHz	0.65	-0.06	0.33	-0.17	-0.38
IPD_HL_MOD_2kHz	0.57	0.27	-0.29	0.55	-0.01
IPD_HL_MOD_0.6kHz	0.56	0.43	-0.18	0.14	-0.10
IPD_HL_PT	0.66	0.46	-0.15	-0.26	-0.01
IPD_LL_MOD_2kHz	0.49	0.56	-0.19	0.48	0.08
IPD_LL_MOD_0.6kHz	0.59	0.40	0.03	-0.29	-0.28
IPD_LL_PT	0.64	0.50	-0.11	-0.23	-0.08

The patterns of loadings for the electrophysiological PCs are shown in Table 3. elec_PC1, which accounted for 31% of the variance, had positive loadings on all the electrophysiological tests. The loadings were high on FFR ENV measures at 600 Hz, moderate on FFR ENV measures at 2 kHz and FFR TFS measures, and generally low on ABR measures. elec_PC2, which accounted for 19% of the variance, had generally high positive loadings on the FFR ENV measures at 2 kHz, moderate negative loadings on the FFR ENV measures at 600 Hz, and small loadings on the other measures. Overall this component appears to reflect variance differentiating the FFR ENV measures at 2 kHz and at 600 Hz. elec_PC3, which accounted for 10% of the variance, had moderate positive loadings on the FFR TFS measures, generally moderate positive loadings on the ABR measures, and small negative loadings on the FFR ENV measures. elec_PC4, which accounted for 9% of the variance, had moderate positive loadings on the ABR measures, except those for wave V at the low stimulus level for which the loadings were positive but low. This component had moderate negative loadings on the FFR TFS measures, and loadings close to zero on the FFR ENV measures. Overall this component seems to reflect variance differentiating the ABR from the FFR TFS measures.

**Table 3:** Loadings (correlations) between PCs and variables of the PCA of electrophysiological measures. For brevity only the results for the first five PCs are shown.

	PC1	PC2	PC3	PC4	PC5
ABR_HL_I_ERL	0.10	0.23	0.31	0.51	0.09
ABR_HL_I_TPR	0.12	0.20	0.49	0.38	0.04
ABR_HL_V_ERL	0.32	0.15	0.34	0.60	0.38
ABR_HL_V_TPR	0.14	0.21	0.26	0.44	0.39
ABR_LL_I_ERL	0.30	-0.10	0.39	0.52	0.09
ABR_LL_I_TPR	0.37	-0.19	0.39	0.40	0.17
ABR_LL_V_ERL	0.27	0.07	0.37	0.22	-0.66
ABR_LL_V_TPR	0.25	0.02	0.38	0.13	-0.70
FFR_ENV_MI0.7_2kHz_C7	0.53	0.63	-0.08	-0.10	-0.13
FFR_ENV_MI0.7_2kHz_ERL	0.46	0.78	-0.20	-0.13	0.18
FFR_ENV_MI0.7_2kHz_MST	0.52	0.51	0.04	0.16	-0.26
FFR_ENV_MI0.7_2kHz_TPR	0.37	0.81	-0.21	-0.10	0.18
FFR_ENV_MI0.7_0.6kHz_C7	0.80	-0.43	-0.21	0.08	-0.12
FFR_ENV_MI0.7_0.6kHz_ERL	0.83	-0.39	-0.22	0.04	0.04
FFR_ENV_MI0.7_0.6kHz_MST	0.71	-0.39	-0.17	0.13	-0.03
FFR_ENV_MI0.7_0.6kHz_TPR	0.82	-0.36	-0.29	0.04	0.02
FFR_ENV_MI1_2kHz_C7	0.59	0.65	-0.07	-0.06	-0.15
FFR_ENV_MI1_2kHz_ERL	0.50	0.77	-0.16	-0.07	0.11
FFR_ENV_MI1_2kHz_MST	0.52	0.54	0.04	0.08	-0.30
FFR_ENV_MI1_2kHz_TPR	0.43	0.82	-0.18	-0.07	0.11
FFR_ENV_MI1_0.6kHz_C7	0.80	-0.45	-0.19	0.06	-0.06
FFR_ENV_MI1_0.6kHz_ERL	0.83	-0.37	-0.26	0.06	0.09
FFR_ENV_MI1_0.6kHz_MST	0.71	-0.42	-0.17	0.10	0.03
FFR_ENV_MI1_0.6kHz_TPR	0.82	-0.34	-0.30	0.06	0.06
FFR_TFS_C7	0.48	-0.06	0.51	-0.50	0.21
FFR_TFS_ERL	0.53	-0.12	0.57	-0.55	0.10
FFR_TFS_MST	0.49	-0.20	0.57	-0.47	0.13
FFR_TFS_TPR	0.57	-0.08	0.54	-0.52	0.03

### 3.2 Relation between speech reception and electrophysiological measures

The relation between speech reception and electrophysiological measures was assessed via an MLR model with the speech-reception PC as the dependent variable, and the four electrophysiological PCs as predictors. The additional control covariates in the model were age, audiometric thresholds (PTA_0.125-2_), lifetime noise exposure, and musical experience.

**Fig. 1:**
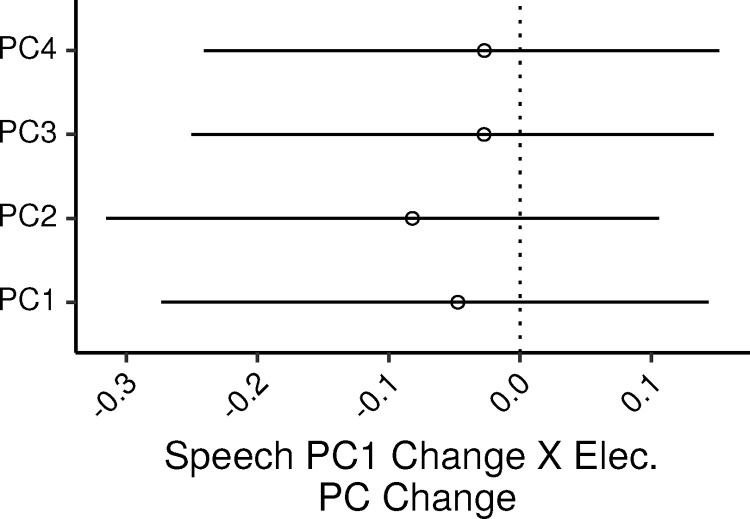
Posterior medians (circles) and 99% CIs for the effects of the electrophysiological measures PCs on the speech-reception PC estimated by the Bayesian MLR model. Effects are plotted as standardized regression coefficients.

Overall the model accounted for 24% of the variance in the dependent measure (CI: 1 – 35%). Fig. 1 shows the 99% CIs for the standardized regression coefficients (ζs) of the four electrophysiological PCs. There was a weak trend for speech_PC1 to decrease with increases in elec_PC2 (ζ CI: -0.31 – 0.1), but none of the ζs were credibly different from zero, with the CIs for the other ζs ranging from ∼−0.27 to ∼0.15. In SLRs of speech_PC1 by each electrophysiological PC, the PVs explained by the PCs were, 2.8, 0.008, 0.28, and 0.3 percent; the UPVs explained by the electrophysiological PCs in the MLR model were, 0.3, 1.3, 0.1, and 0.1 percent.

### 3.3 Relation between speech reception and psychophysical measures of temporal processing

The relation between speech reception and psychophysical measures was assessed via an MLR model with the speech-reception PC as the dependent variable, and the two psychophysical PCs as predictors. The additional control covariates in the model were age, audiometric thresholds (PTA_0.125-2_), lifetime noise exposure, cognitive abilities, and musical experience.

**Fig. 2:**
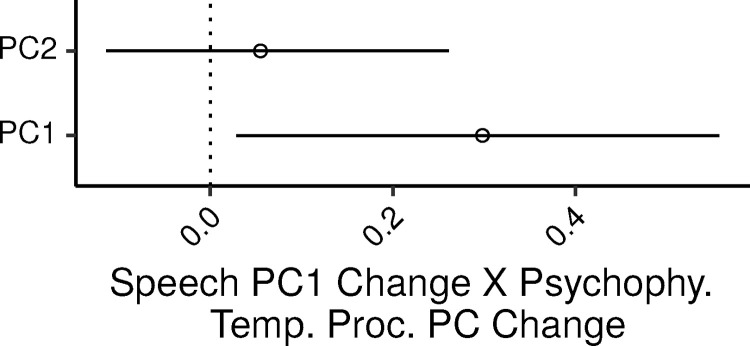
Posterior medians (circles) and 99% CIs for the effects of the psychophysical measures of temporal processing PCs on the speech-reception PC estimated by the Bayesian MLR model. Effects are plotted as standardized regression coefficients.

Overall the model accounted for 32% of the variance in the dependent measure (CI: 19 – 46%). Fig. 2 shows the 99% CIs for the ζs of the two psychophysical temporal processing PCs. There was a credible increase in speech_PC1 with increasing psyphy_PC1, with the posterior median of ζ equal to 0.3 (CI: 0.03 – 0.56). Thus, relative to the variance captured by the two PCs, worse speech-reception thresholds were associated with worse thresholds in the psychophysical tasks, independently of the effect of the covariates. In an SLR of speech_PC1 by psyphy_PC1 the PV explained by this variable was 22%; the UPV explained by psyphy_PC1 in the MLR model was instead 7%. The association between speech_PC1 and psyphy_PC2 was not credibly different from zero (ζ CI: -0.11 – 0.26; SLR PV: 1%; MLR UPV: 0.5%).

### 3.4 Relation between consonance preference and electrophysiological measures

The relation between consonance preference and electrophysiological measures was assessed via an MLR model with the consonance preference PC as the dependent variable, and the four electrophysiological PCs as predictors. The additional control covariates in the model were age, audiometric thresholds (PTA_0.125-2_), lifetime noise exposure, and musical experience.

Overall the model accounted for 12% of the variance in the dependent measure (CI: 2.4 – 21.7%).

**Fig. 3:**
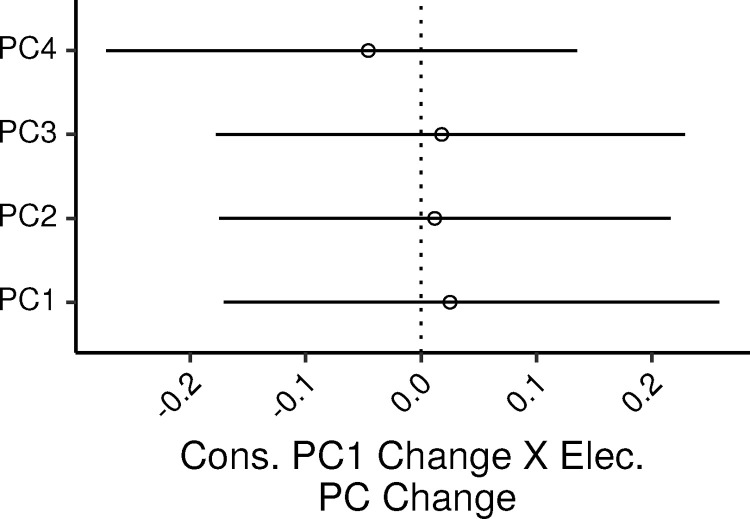
Posterior medians (circles) and 99% CIs for the effects of the electrophysiological measures PCs on the consonance preference PC estimated by the Bayesian MLR model. Effects are plotted as standardized regression coefficients.

Fig. 3 shows the 99% CIs for the standardized regression coefficients (ζs) of the four electrophysiological PCs. None of the ζs were credibly different from zero, with the CIs ranging from ∼−0.27 to ∼0.26. In SLRs of cons_PC1 by each electrophysiological PC, the PV explained by the PCs were 1.34, 0.04, 0.12, and 0.37 percent; the UPV explained by the electrophysiological PCs in the MLR model were 0, 0.06, 0.06, and 0.4 percent.

### 3.5 Relation between consonance preference and psychophysical measures of temporal processing

The relation between consonance preference and psychophysical measures was assessed via an MLR model with the consonance preference PC as the dependent variable, and the two psychophysical PCs as predictors. The additional control covariates in the model were age, audiometric thresholds (PTA_0.125-2_), lifetime noise exposure, cognitive abilities, and musical experience.

Overall the model accounted for 24% of the variance in the dependent measure (CI: 12 – 36%).

**Fig. 4:**
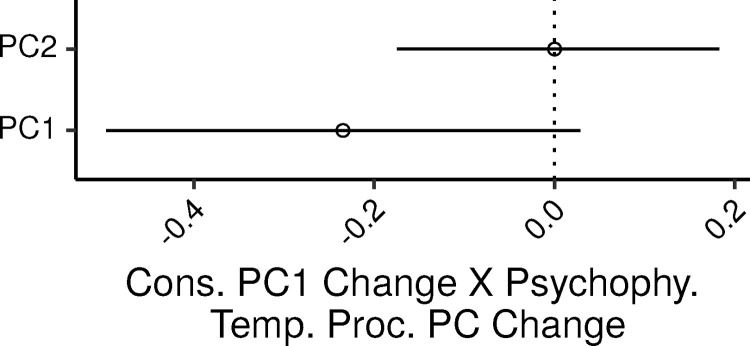
Posterior medians (circles) and 99% CIs for the effects of the psychophysical measures of temporal processing PCs on the consonance preference PC estimated by the Bayesian MLR model. Effects are plotted as standardized regression coefficients.

Fig. 4 shows the 99% CIs for the ζs of the two psychophysical temporal processing PCs. There was a trend for cons_PC1 to decrease with increasing psyphy_PC1, reflecting a trend for reduced consonance preference for participants with higher thresholds in the psychophysical temporal processing tests. The posterior median ζ of this effect was -0.23 (CI: -0.5 – 0.03). In an SLR of cons_PC1 by psyphy_PC1 the PV explained by this variable was 16%; the UPV explained by psyphy_PC1 in the MLR model was instead 4.3%. The association between cons_PC1 and psyphy_PC2 was not credibly different from zero (ζ CI: -0.17 – 0.18; SLR PV: 0.001%; MLR UPV: 0.009%).

### 3.6 Relation between psychophysical measures of temporal processing and electrophysiological measures

**Fig. 5:**
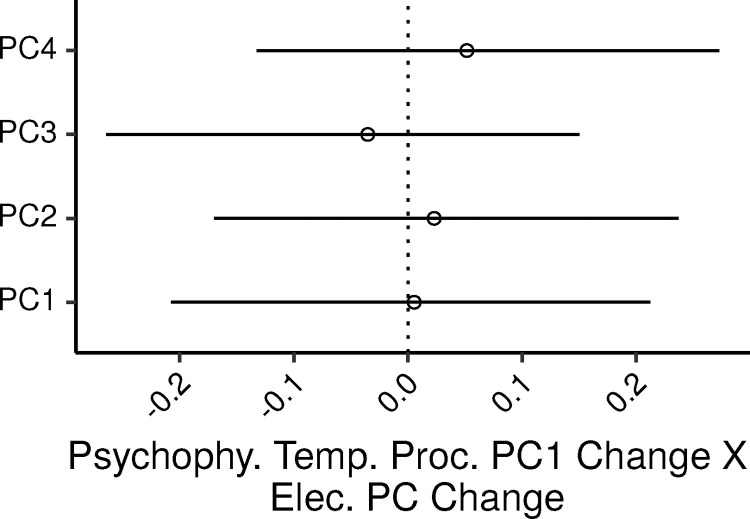
Posterior medians (circles) and 99% CIs for the effects of the electrophysiological measures PCs on the psychophysical measures of temporal processing PC1 estimated by the Bayesian MLR model. Effects are plotted as standardized regression coefficients.

The relation between psychophysical measures of temporal processing and electrophysiological measures was assessed via two MLR models, one with psyphy_PC1, and one with psyphy_PC2 as the dependent variable, and the four electrophysiological PCs as predictors. The additional control covariates in the model were age, audiometric thresholds (PTA_0.125-2_), lifetime noise exposure, and musical experience. Overall the model for psyphy_PC1 accounted for 17% of the variance in the dependent measure (CI: 6 – 29%). Fig. 5 shows the 99% CIs for the standardized regression coefficients of the four electrophysiological PCs. None of the ζs were credibly different from zero, with CIs ranging from ∼−0.26 to ∼0.27. In SLRs of psyphy_PC1 by each electrophysiological PC, the PV explained by the PCs were, 0.9, 1.2, 0.9, and 0.2 percent; the UPV explained by the electrophysiological PCs in the MLR model were 0.03, 0, 0.14, and 0.65 percent.

Overall the model for psyphy_PC2 accounted for 3% of the variance in the dependent measure (CI: 0 – 7.6%).

**Fig. 6:**
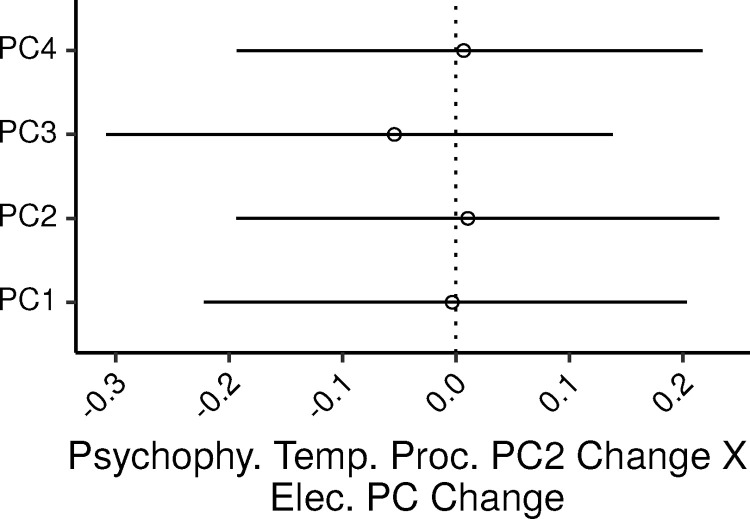
Posterior medians (circles) and 99% CIs for the effects of the electrophysiological measures PCs on the psychophysical measures of temporal processing PC2 estimated by the Bayesian MLR model. Effects are plotted as standardized regression coefficients.

Fig. 6 shows the 99% CIs for the standardized regression coefficients of the four electrophysiological PCs. None of the ζs were credibly different from zero, with CIs ranging from ∼−0.22 to ∼0.23. In SLRs of psyphy_PC2 by each electrophysiological PC, the PV explained by the PCs were 0.03, 0.09, 0.51, and 0.02 percent; the UPV explained by the electrophysiological PCs in the MLR model were 0, 0.01, 0.6, and 0.01 percent.

### 3.7 Exploratory analyses

#### 3.7.1 Relation of behavioral measures with auditory brainstem responses in quiet

The main analyses did not include the ABR in quiet (ABRQ) measures because these measures are affected by high-frequency cochlear regions that did not contribute to stimulus coding in the behavioral measures. However, it is possible that even if these high-frequency cochlear regions did not directly contribute to stimulus coding, their physiological status was indirectly associated with physiological dysfunctions in low-frequency cochlear regions that were perhaps not captured by the frequency-specific electrophysiological responses. For this reason we performed additional analyses looking at the associations between both real-world measures of hearing abilities, and psychophysical measures of temporal processing, with the ABR in quiet responses.

The ABR in quiet responses were first subject to a PCA using the same methodology employed for the other measures. Three PCs were extracted; overall they accounted for 75% of the variance (PC1: 37%; PC2: 22%, PC3: 15%, see Table S7). The first PC had moderate/high positive loadings on all the variables (see Table S8). The second PC had moderate positive loadings on the wave I measures and small/moderate negative loadings on the wave V measures. The third PC tended to have positive loadings on the low-level measures (moderate for wave I and small for wave V), and negative loadings on the high-level measures (moderate for wave I and small for wave V).

The ABR in quiet PCs were then used, in separate models, as predictors of the speech reception PC, of the consonance preference scores PC, and of the psychophysical temporal processing PCs, using MLR models similar to those employed for assessing the associations between these behavioral measures and the electrophysiological responses in noise. The only difference between the models used for the electrophysiological responses in high-pass masking noise and those used for the ABR in quiet was the addition of a further covariate (average PTA between 4 and 12 kHz; PTA_4__–__12_) in the ABR in quiet models to control for effects of high-frequency audiometric thresholds shifts.

The results of the ABR in quiet models are shown in Figure S1. None of these models revealed credible effects of the ABR in quiet PCs on the behavioral measures. The CIs for the models using as dependent variables the speech-reception PC, and the first psychophysical temporal processing PC were relatively narrow (∼±0.2). For the consonance preference model there were weak trends for the consonance preference PC to decrease with increases in the ABR in quiet PC1 and PC2 (CIs ∼ -0.3 – 0.1). For the model using as dependent variable the second psychophysical temporal processing PC there were trends for this variable to decrease with increases in all ABR in quiet PCs (CIs ∼ -0.4 – 0.1).

Overall, while the analyses did not find evidence of associations between behavioral measures and ABR in quiet measures, they are not inconsistent with the possibility that moderate associations exist between both the consonance preference PC and the second psychophysical temporal processing PC and some of the ABR in quiet PCs.

#### 3.7.2 Principal component analyses of difference measures and relations between difference measures

Our test battery contained measures obtained at high and at low stimulus levels (or, for the FFR, measures obtained with a shallow and with a deep modulation amplitude) that should engage differentially auditory nerve fibers with low/medium spontaneous rate, which are thought to be most affected by noise-induced (Furman et al., 2013), and age-related CS in rodents (see discussion in Carcagno and Plack, 2021). In our previous studies (Carcagno, Plack, 2020, Carcagno, Plack, 2021) we did not find effects of age on most of these differential measures (the only exception was for AMD at 50 Hz) for stimuli restricted (by filtering and highpass masking) in a low frequency region where thresholds were relatively well preserved across the age range. We found effects of age consistent with specific deficits of L/M-SR fibers on ABR amplitudes obtained in quiet, that reflect the contribution of higher cochlear frequency regions than those coding for the stimuli used in the real-world and psychophysical temporal processing tests employed in the current study. Additionally, we did not find effects of lifetime noise exposure (estimated with a restrospective questionnaire) on any of these differential measures. The lack of consistent effects of age or lifetime noise exposure on these differential measures does not suggest the presence of systematic relations between them. Additionally, the fact that the PCs of the raw measures reported in the current paper did not tend to load differentialy to high/low level or deep/shallow modulation stimuli, suggests that there was little systematic variance related to these differential measures across tasks. Nonetheless it is possible that the PCAs on the raw measures missed some smaller variance components related to the differential measures, and that some of these measures are related to each other. For exhaustiveness, to assess this possbility, we performed the analyses that will be described in this section.

For the speech and psychophysical temporal processing tests the difference measures were derived by subtracting thresholds obtained at the low stimulus levels from those obtained at the high stimulus levels. A higher score on the differential measure thus reflects worse performance at the high stimulus level (*re* performance at the low stimulus level).

The consonance preference differential measure was derived by subtracting preference scores obtained at the low stimulus level from those obtained at the high stimulus level. The ABR amplitude difference measures were similarly derived by subtracting wave amplitudes obtained at the low stimulus level from those obtained at the high stimulus level. The FFR signal-to-noise ratio (SNR) difference measures were derived by subtracting SNR values obtained at the deep modulation depth from those obtained at the shallow modulation depth. For all these measures (consonance preference, ABR amplitudes, and FFR SNR) a lower score on the differential measure reflects a “worse” outcome for the stimulus with a high level or a shallow modulation depth (*re* outcome for the stimulus with a low level or a deep modulation depth).

As for the “raw” (non-differential) measures presented before, a series of PCAs were used to extract the major variance components from each test domain. These PCs will be denoted by the “diff” prefix to distinguish them from the “raw” PCs presented before. For the speech tests, parallel analysis suggested that no common variance components could be extracted. This indicates that the differential measures for the three speech tests either correlated very weakly or were uncorrelated. Each speech test was thus treated as a separate variable in the following analyses, without further data reduction. There was only one differential measure of consonance preference, thus also in this case no further data reduction was possible. The PCA results for the other domains are presented in the SM. For the psychophysical measures of temporal processing parallel analysis indicated the extraction of two components accounting for 39% of the variance. For the electrophysiological measures parallel analysis indicated the extraction of four components accounting for 68% of the variance. A separate PCA was also performed on the ABR in quiet measures, for which parallel analysis indicated the extraction of two components, accounting for 76% of the variance.

As for the non-differential measures a series of Bayesian MLR models were used to assess the relations between the PCs of the differential measures (or the simple differential measures in the case of the speech and consonance preference tests) in different domains. The models aimed to assess: i) the relations between speech and electrophysiological (excluding ABR in quiet) difference measures, ii) the relations between speech and psychophysical temporal processing difference measures, iii) the relations between consonance preference and electrophysiological (excluding ABR in quiet) difference measures, iv) the relations between consonance preference and psychophysical temporal processing difference measures, and v) the relations between psychophysical temporal processing and electrophysiological (excluding ABR in quiet) difference measures. Additional MLR models were run to assess the relations between a) speech b) consonance preference and c) psychophysical temporal processing difference measures with the ABR in quiet difference measures. All models included as covariates age, PTA_0.125-2_, and lifetime noise exposure. The models using as predictors the ABR in quiet PCs additionally included PTA_4__–__12_ as a covariate. In total 16 MLR models were run to assess the relations between the difference measures (see Table S15).

The results of the MLR models are shown in Figures S2, S3 and S4, and will be described succinctly. In all these models, the only effect of interest that was credibly different from zero was a decrease in diff_psyphy_PC1 with increasing diff_ABRQ_PC2 (ζ CI: -0.57 – -0.09). Given that diff_psyphy_PC1 loaded mainly on the AMD tests and diff_ABRQ_PC2 mainly loaded on the wave V amplitudes (see SM), this result suggests that worse performance at the high stimulus level in the AMD tests was associated with smaller wave V amplitudes in quiet at the high stimulus level.

A few other effects showed relatively defined trends, but were not credibly different from zero. diff_CRM_OFF tended to increase with increases in both diff_psyphy_PC1 and diff_psyphy_PC2. diff_DTT also tended to increase with increases in diff_psyphy_PC1. Thus, worse performance at the high stimulus level in some of the speech tests tended to be associated with worse performance at the high stimulus level in the psychophysical temporal processing tests. diff_DTT tended to decrease with increasing diff_elec_PC3. Given that diff_elec_PC3 mainly reflected wave I ABR amplitudes (in HP noise), this result suggests that worse performance at the high stimulus level in the DTT task tended to be associated with smaller wave I amplitudes at the high stimulus level.

Increases in diff_elec_PC4 tended to be associated with decreases in diff_cons. diff_elec_PC4 mainly loaded on the ABR wave V amplitudes but the pattern of loadings for this PC was quite complex and included smaller positive and negative loadings on some FFR measures. Overall, this result suggests that higher wave V amplitudes for the high level stimuli tended to be associated with lower consonance preference scores at the high stimulus level.

## 4 Discussion

In this study we assessed the relations between a large set of behavioral and electrophysiological measures that were collected for an investigation of age-related hearing declines on 102 listeners across the age range (Carcagno, Plack, 2020, Carcagno, Plack, 2021). In order to deal with the issue of the large number of variables assessed, some of which measured the same constructs, we used PCA to reduce the dimensionality of the dataset while preserving the largest sources of variance within the different domains to which the variables belonged (speech reception, consonance preference, psychophysical temporal processing, and electrophysiological subcortical function measures). We found that increases (reflecting worse thresholds) in the first PC of the psychophysical temporal processing measures were credibly associated with increases (reflecting worse thresholds) of the first speech-reception PC independently of age, low-frequency audiometric thresholds, lifetime noise exposure, cognitive abilities, and musical experience. Increases in the first psychophysical temporal processing PC tended to be also associated with decreases (reflecting reduced consonance preference) in the first consonance preference PC, although this association was not credibly different from zero. Electrophysiological PCs (which were derived from several ABR and FFR measures), however, were not found to be associated with either speech reception, consonance preference, or psychophysical temporal processing PCs. The credibility intervals for these effects were generally narrow, indicating that, even if such associations did exist (but were not detected in the current study), they would be small. Our findings will be discussed in reference to other studies that have investigated the relations between speech reception, psychophysical temporal processing, and electrophysiological measures similar to those employed in the current study, and on participants with similar characteristics (in particular normal or near-normal audiometric thresholds in the frequency region coding for the stimuli).

Before discussing these results, it is important to point out that the reason why few sizeable associations were found in the current study is not linked to the choices of using PCA to compress the dimensionality of the dataset and of selecting only a restricted number of components, as suggested by parallel analysis. Further exploratory analyses (not reported in the paper for brevity) in which all PCs explaining at least 10% of the variance were included did not reveal any new associations. Also, inspection of the correlation matrix between the different measures, shown in Table S16, does not suggest that testing each individual variable instead of compressing the dataset using PCA would have led to discovering other sizeable associations.

The finding of a relation between speech-reception and psychophysical temporal processing measures is consistent with the results of Füllgrabe et al. (2014), Schoof and Rosen (2014), and Oberfeld and Klöckner-Nowotny (2016). Several methodological differences between the studies (e.g. the use of closed-set speech tests vs open-set speech tests, the different tests used to measure auditory temporal processing, the inclusion of different control variables, or the use of different tests to measure the same confounders) prevent meaningful quantitative comparisons of their results. However, it is interesting to point out some of the differences between these studies. Füllgrabe et al. (2014) found that both consonant identification in bisyllabic vowel-consonant-vowel stimuli presented in noise, and identification of target sentences taken from the corpus of the Adaptive Sentence Lists (MacLeod and Summerfield, 1990) presented in two-talker babble, were significantly predicted by a composite measure of TFS sensitivity [TFS1 (Moore and Sek, 2009) and TFS-LF (Hopkins and Moore, 2010) tests], while this was not the case when temporal ENV sensitivity (assessed by AMD thresholds) was used as a predictor.

Schoof and Rosen (2014) found that frequency modulation (FM) detection thresholds, which reflect TFS processing, significantly predicted speech-reception thresholds for IEEE sentences (Rothauser et al., 1969) in two-talker babble, while this was not the case for speech-reception thresholds in noise. However, the slopes of the effect of FM detection thresholds on speech reception thresholds were not significantly different between the models for speech reception in babble and in noise. Two other measures, AMD and gap detection thresholds, which reflect temporal ENV processing, were not selected as significant predictors by the best subset regression models used for the analyses.

Oberfeld and Klöckner-Nowotny (2016) found TFS-LF thresholds to significantly predict the identification of sentences spoken by a target talker from sentences spoken by two interfering talkers offset by ±25 degrees.

The results of Füllgrabe et al. (2014) and Schoof and Rosen (2014) are suggestive of a greater importance of TFS compared to ENV information (measured using AMD thresholds) for speech reception. However, neither of the two studies made a direct comparison showing that TFS information has a *greater* effect on speech-reception thresholds than ENV information. Moreover, in our study the first psychophysical temporal processing PC, which was credibly associated with the first speech-reception PC, had its highest loadings on the AMD tasks, which reflects temporal ENV processing. The results of Schoof and Rosen (2014) are also suggestive of a greater importance of temporal information for the reception of speech in the presence of interfering speech compared to the reception of speech in the presence of noise, but as pointed out by the authors, the lack of a significant difference between the slopes of the effects for the two types of interferers (speech or noise) does not provide evidence to support this notion. Our data cannot conclusively address this issue because different types of maskers (noise or speech) were used for different tests (DTT or CRM), thus the possible effect of masker type is confounded with that of test type. Despite this limitation, we performed a supplementary exploratory analysis (not reported in the manuscript), in which the DTT (which used a noise masker), and the CRM (which used interfering talkers) thresholds were averaged across the different task conditions to obtain a single DTT threshold and a single CRM threshold. The DTT and CRM thresholds were then analyzed within a single MLR model similar to that used for the speech PCA scores, but with an additional dummy variable indicating the test type and random subject effects to account for the correlated within-subjects measures. The results revealed that psyphy_PC1 was a credible predictor of CRM thresholds, while this was not the case for DTT thresholds. As in Schoof and Rosen (2014), however, the difference between the slopes of the effects for the two types of interferers was not credibly different from zero. Therefore, while our results are suggestive of a greater importance of TFS information for speech masked by interfering speech than for speech masked by noise, they do not provide conclusive evidence for this.

Overall, the results of the studies described above, including the current one, show that an association between temporal processing, measured psychophysically, and speech reception in the presence of interfering talkers, can be observed under a variety of different test measures and methodologies. It remains unclear whether TFS information is more strongly associated with speech reception compared to ENV information, or whether the effect of temporal information is more important for the reception of speech masked by competing speech compared to the reception of speech masked by noise.

An issue with the interpretation of the association between speech-reception and psychophysical temporal processing measures in the current study, as well as in the previous ones, is that, while the tests employed to assess psychophysical temporal processing abilities are thought to rely to a large extent on such abilities, they may also rely on other general auditory abilities needed to perform psychoacoustics discrimination tasks, and none of these studies provided evidence that the association is specific for psychophysical *temporal* processing tests.^1^ Further studies will be needed to assess the specificity of this association.

The lack of credible associations between speech reception and electrophysiological subcortical auditory function measures found in the current study is consistent with a number of previous studies that also failed to find such associations (Bramhall, Konrad-Martin, McMillan, 2018, Guest, Munro, Prendergast, Millman, Plack, 2018, Johannesen, Buzo, Lopez-Poveda, 2019, Prendergast, Couth, Millman, Guest, Kluk, Munro, Plack, 2019, Prendergast, Millman, Guest, Munro, Kluk, Dewey, Hall, Heinz, Plack, 2017, Schoof, Rosen, 2016, Smith, Krizman, Liu, White-Schwoch, Nicol, Kraus, 2019). However, other studies have reported significant associations between these measures (Liberman, Epstein, Cleveland, Wang, Maison, 2016, Mepani, Kirk, Hancock, Bennett, de Gruttola, Liberman, Maison, 2020, Valderrama, Beach, Yeend, Sharma, Van Dun, Dillon, 2018). The reasons for the discrepancies between the results of these studies are unclear, and hard to pinpoint due to the different tests and measures used to assess both speech reception, and electrophysiological variables. It is possible that some of the specific electrophysiological measures used in these studies were not sufficiently sensitive to the neurophysiological subcortical processes that could be constraining performance and explaining variability in the speech-reception tasks. In this respect, it has been argued that electrocochleography measures, and in particular the ratio of the action potential (AP; which corresponds to the ABR wave I peak) to the summating potential (SP; an inflection point on the rising side of the AP) may be more sensitive (Grant, Mepani, Wu, Hancock, de Gruttola, Liberman, Maison, 2020, Mepani, Kirk, Hancock, Bennett, de Gruttola, Liberman, Maison, 2020). Two (Liberman, Epstein, Cleveland, Wang, Maison, 2016, Mepani, Kirk, Hancock, Bennett, de Gruttola, Liberman, Maison, 2020) out of the three above-mentioned studies finding associations between speech-reception and subcortical neural processing measures used the AP/SP ratio to assess subcortical neural processing. The interpretation of these results, however, is unclear because the effect has been found to depend mainly on changes of the SP, which has traditionally been thought to originate from inner hair cell receptor potentials rather than from neural elements (Durrant, Wang, Ding, Salvi, 1998, Eggermont, 2017).^2^ Some recent studies, however, suggest that the SP may include neural components (Kennedy, Kaf, Ferraro, Delgado, Lichtenhan, 2017, Pappa, Hutson, Scott, Wilson, Fox, Masood, Giardina, Pulver, Grana, Askew, Fitzpatrick, 2019). The interpretation of the association between the AP/SP ratio and speech-reception thresholds thus hinges on the interpretation of the SP, which has not yet been fully elucidated.

Another possible explanation of the inconsistent findings re associations between subcortical neural processing and speech-reception measures is that, at least for subjects with relatively normal hearing from the general population (most of which likely did not have any neurological deficits) the amount of variance in speech-reception performance explained by subcortical neural processing is so low, that even studies testing a large number of participants, like the current one, cannot consistently find an association between the two measures. Given that speech is a highly complex stimulus, and its perception involves a wide network of cortical areas (Peelle, 2019), it is not implausible that variance in speech-reception thresholds in normal-hearing and neurologically healthy subjects may show little dependence on variance in subcortical neural processing.

Although we have so far limited the discussion to studies employing stimuli similar to those used in the current study, it is worth noting that several studies using more complex speech stimuli to elicit electrophysiological responses, such as syllables, have found significant associations between these electrophysiological responses and masked-speech reception (Anderson, Parbery-Clark, Yi, Kraus, 2011, Bidelman, Momtaz, 2021, Ruggles, Bharadwaj, Shinn-Cunningham, 2011, Ruggles, Bharadwaj, Shinn-Cunningham, 2012, Song, Skoe, Banai, Kraus, 2011). The reason why associations between masked-speech reception and electrophysiological measures seem to be more consistently reported when speech stimuli are used to elicit the electrophysiological responses, compared to more basic non-speech stimuli, is unclear. One possibility is that speech stimuli engage to a greater extent top-down corticofugal pathways (Chandrasekaran et al., 2014) whose functioning may better relate to masked-speech reception. Although these studies using speech stimuli to evoke electrophysiological responses were run on normal-hearing listeners, or attempted to account for variations in hearing sensitivity statistically, it is difficult to exclude the possibility that sub-clinical hair-cell damage may have contributed to the observed associations. Statistically controlling for variations in hearing sensitivity becomes difficult when wide cochlear regions are stimulated (Carcagno and Plack, 2020). Clearer evidence of associations between masked-speech reception and subcortical electrophysiological measures could be obtained by using highpass masking techniques (Nuttall et al., 2015) similar to those employed in the current study to better delimit the cochlear sources of the electrophysiological and behavioral responses.

The lack of relations between psychophysical measures of auditory temporal processing and electrophysiological measures appears inconsistent with the results of some other studies using qualitatively similar measures that have found such relations (Bharadwaj, Masud, Mehraei, Verhulst, Shinn-Cunningham, 2015, Verhulst, Ernst, Garrett, Vasilkov, 2018), but is in line with the results of another large scale study (Prendergast et al., 2017). The reasons for these discrepancies remain unclear. Further exploratory analyses (not reported in the paper) in which we tried to approximate statistical tests similar to those employed by Bharadwaj et al. (2015) by assessing the relation between the FFR ENV difference measure at 2 kHz and IPD MOD 2 kHz thresholds at the high stimulus level, and the relations between the FFR ENV difference measure at 2 kHz and AMD at 25 or at 100 Hz at the high stimulus level (in each case while partialing out the effects of age and PTA_0.125-2_) failed to find evidence of associations between these measures in our study. However, it should be noted that even though we tried to select tests similar to those employed by Bharadwaj et al. (2015) from our test battery, several methodological differences between the studies remain. Overall, the results of Prendergast et al. (2017) and those of the current study suggest that performance in psychophysical temporal processing tasks is not limited by neural processing abilities, as indexed by the electrophysiological measures used in these studies, within the range found in listeners with relatively good low-frequency thresholds. It remains unclear whether this may be due to the fact that the electrophysiological measures used are not diagnostic of the neural processing abilities actually limiting performance in the tasks, or whether these neural processing abilities are sufficiently good across the population tested as to not constitute a performance bottleneck. This latter possibility is consistent with some models predicting negligible impacts of even major losses of auditory nerve synapses on psychophysical discrimination performance (Oxenham, 2016).

A limitation of the current study is that the ABR and FFR latency measures could not be included because of missing latency data points that could not be imputed in a straightforward way. Running the analyses ignoring the issue either by leaving out the missing data points, or imputing them by assuming that they are missing at random, risks biasing the results. While the proportion of missing data points for the FFR measures was relatively high (28% for ENV and 75% for TFS), for the ABR waves in high-pass masking noise the missing data points were relatively few (2.9%), and one may be willing to ignore the issue. In an additional analysis (not reported in the manuscript) we imputed the missing ABR wave latency data points (obtained in high-pass masking noise) using mean values, ran a PCA on the ABR wave latency values which resulted in three PCs, and used these PCs as independent variables in multiple regression models similar to those used for ABR amplitudes to explain variance in the speech, psychophysical temporal processing, and consonance preference PCs. In none of the models were the wave latency PCs credibly related to the speech, psychophysical temporal processing, or consonance preference PCs. Although the results of this additional analysis should be evaluated with caution, due to the missing data issue, they do not provide evidence that ABR in high-pass masking noise wave latencies have relations with the speech, psychophysical temporal processing, or consonance preference measures of the current study.

One major difference between the current study and previous studies investigating the relations between subcortical electrophysiological and speech-reception measures and between subcortical electrophysiological and psychophysical temporal processing measures is that the potential confounding effect of high-frequency sensitivity was strictly controlled for in the current study through the use of highpass masking noise. Both ABR wave I (Don, Eggermont, 1978, Eggermont, Don, 1980) and the FFR (Dau, 2003, Encina-Llamas, Harte, Dau, Shinn-Cunningham, Epp, 2019) have dominant contributions from high-frequency cochlear channels in the absence of highpass noise masking. There is evidence that unfiltered speech contains useful information in the extended high-frequency range and masked-speech reception can be affected by sensitivity in this frequency range (Hunter et al., 2020). Also for psychophysical temporal processing tasks employing low-frequency stimuli, it is possible that at high stimulus levels high-frequency cochlear neurons may be recruited through their low-frequency tails and contribute to performance (Millman and Bacon, 2008). Although some previous studies did control for high-frequency sensitivity statistically, this is likely a suboptimal solution compared to the use of highpass masking noise, because the control variable typically consists of the average threshold over several frequencies that may not all contribute equally to the responses.^3^ Overall, it is possible that lack of control, or suboptimal control of high-frequency sensitivity could account for some of the associations reported previously.

## 5 Conclusions

The results of the current study confirm the presence of an association between psychophysical temporal processing abilities and performance in masked-speech reception tasks. The lack of credible associations between speech-reception and subcortical electrophyisiological processing measures suggests that either the electrophysiological measures employed are not sufficiently sensitive to the subcortical neural processes explaining variance in speech-reception performance, or that variance in speech-reception performance (at least in the population of listeners tested in the current study, with near-normal low frequency thresholds and no obvious neurological deficits) shows little dependence on variance in subcortical neural processing. Similar considerations could explain the lack of credible associations found in the current study between psychophysical measures of auditory temporal processing and subcortical electrophysiological measures, and between consonance preference and subcortical electrophysiological measures.

## Declaration of Competing Interest

The authors declare that they have no conflict of interest.

## Appendix A Supplementary materials

Supplementary Data S1 Supplementary Raw Research Data. This is open data under the CC BY license http://creativecommons.org/licenses/by/4.0/ Supplementary Data S1

## Footnotes

1 Füllgrabe et al. (2014) and Schoof and Rosen (2014) used tests involving either ENV or TFS processing. While their results are suggestive of a greater association of TFS processing with speech reception, no direct comparison was made between the strength of the association of speech-reception performance with TFS and ENV processing abilities.

2 A third study (Grant et al., 2020) found relations between the SP, the difference between the AP and the SP (defined as the AP in the paper), and the AP (measured from peak to through and defined as the AP-P_1_ in the paper) and speech-reception measures. However, in the main analyses of this study, no adjustment for the confounding effects of age were made. The results of a secondary analysis based on stepwise multiple regression suggest the presence of a relation between the AP and some speech-reception measures even after controlling for age, however, these associations would not have survived corrections for multiple comparisons (which are not reported in the paper). Relations between the AP-SP difference and speech reception measures were stronger, however, it should be noted that this measure, like the SP/AP ratio is dependent on the amplitude of the SP.

3 An additional issue is that in some cases, in the presence of correlated control variables such as extended high-frequency thresholds and age, authors have opted to partial out only one of them. While in such cases controlling for one variable would likely remove most of the confounding effect of the other variable, unless the control variables are perfectly or very highly correlated there could still be a residual confound related to variance in the omitted control variable that is not captured by the one included in the model. Given that multicollinearity of control variables does not pose particular issues (Voss, 2005), in such cases it would be desirable to include both control variables in the model.
