# Supplementary material for: Relations between speech‐reception, psychophysical temporal processing, and subcortical electrophysiological measures of auditory function in humans
Source: Hear Res. 2022 Apr;417:108456. doi: 10.1016/j.heares.2022.108456 (PMC8935383; doi:10.1016/j.heares.2022.108456)
Supplement: Supplementary Data S1 — Supplementary Raw Research Data. This is open data under the CC BY license http://creativecommons.org/licenses/by/4.0/ [file mmc1.pdf]

Supplementary Materials for “Relations between speech-reception, psychophysical temporal processing, and subcortical electrophysiological measures of auditory function in humans”

Contents

1 Supplementary figures referenced in the main manuscript **2**

2 Supplementary principal component analyses results **6**

2.1 Principal component analysis of cognitive measures 9
2.2 Principal component analysis of auditory brainstem response in quiet measures 9
2.3 Principal component analysis of psychophysical temporal processing difference measures 11
2.4 Principal component analysis of electrophysiological difference measures 12
2.5 Principal component analysis of auditory brainstem response in quiet difference measures 13

3 Difference measures models **15**

4 Correlations **16**

1 Supplementary figures referenced in the main manuscript

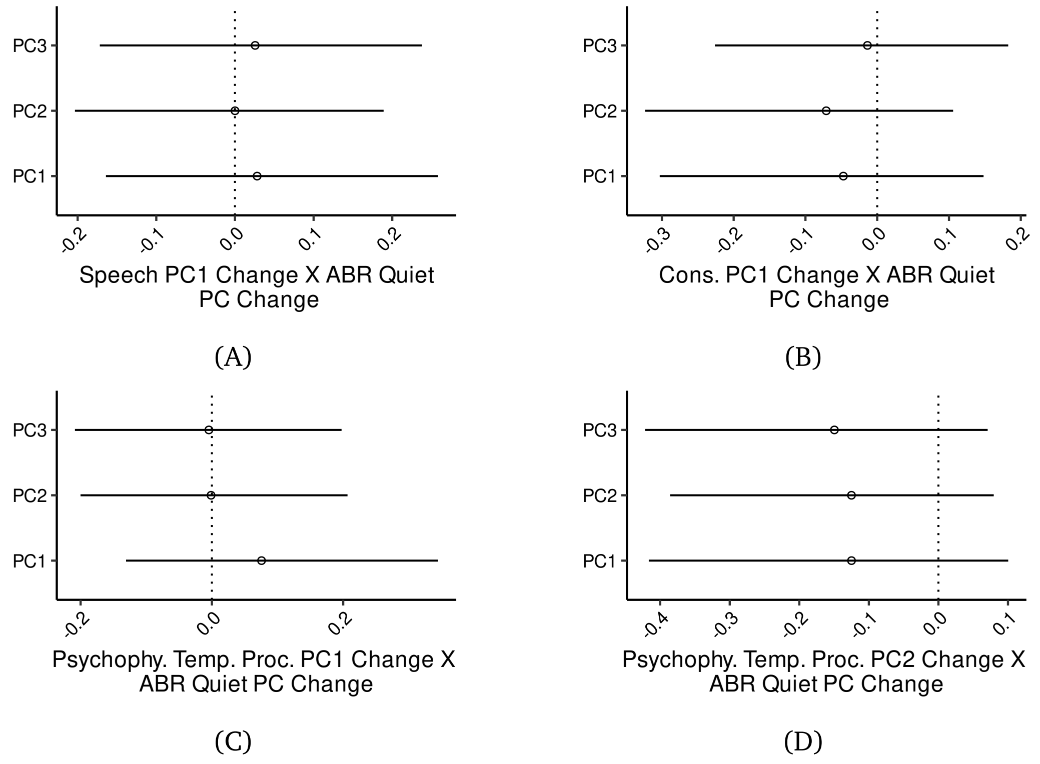

Figure S1: Posterior medians (circles) and 99% CIs for the effects of the ABR in quiet PCs on A) the speech-reception PC1, B) the consonance preference PC1, C) the psychophysical measures of temporal processing PC1, and D) the psychophysical measures of temporal processing PC2 estimated by the Bayesian MLR models. Effects are plotted as standardized regression coefficients.

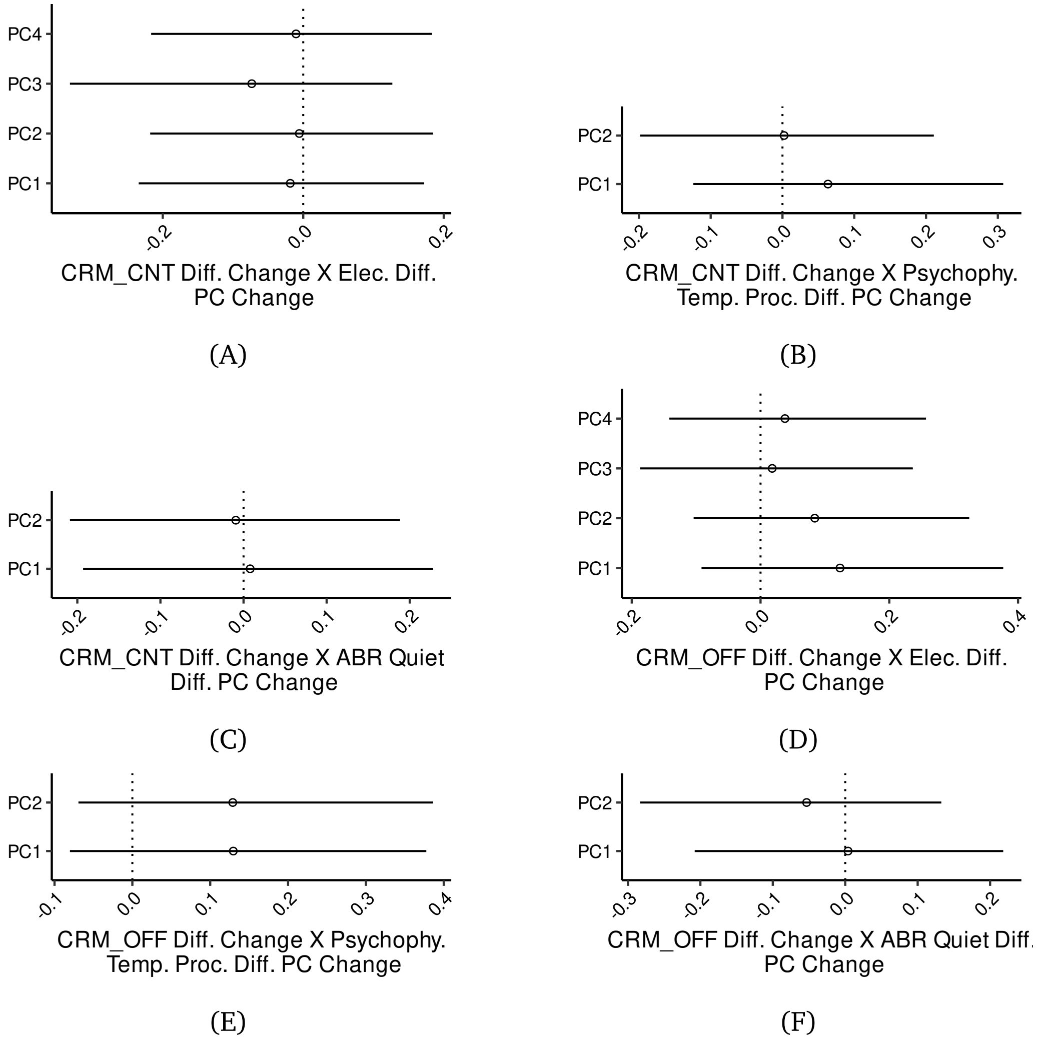

Figure S2: Posterior medians (circles) and 99% CIs for the effects of A) the electrophysiological difference measures (in HP masking noise) PCs on the CRM_CNT difference thresholds, B) the psychophysical temporal processing difference PCs on the CRM_CNT difference thresholds C) the ABR in quiet difference measures PCs on the CRM_CNT difference thresholds, D) the electrophysiological difference measures (in HP masking noise) PCs on the CRM_OFF difference thresholds, E) the psychophysical temporal processing difference PCs on the CRM_OFF difference thresholds F) the ABR in quiet difference measures PCs on the CRM_OFF difference thresholds.

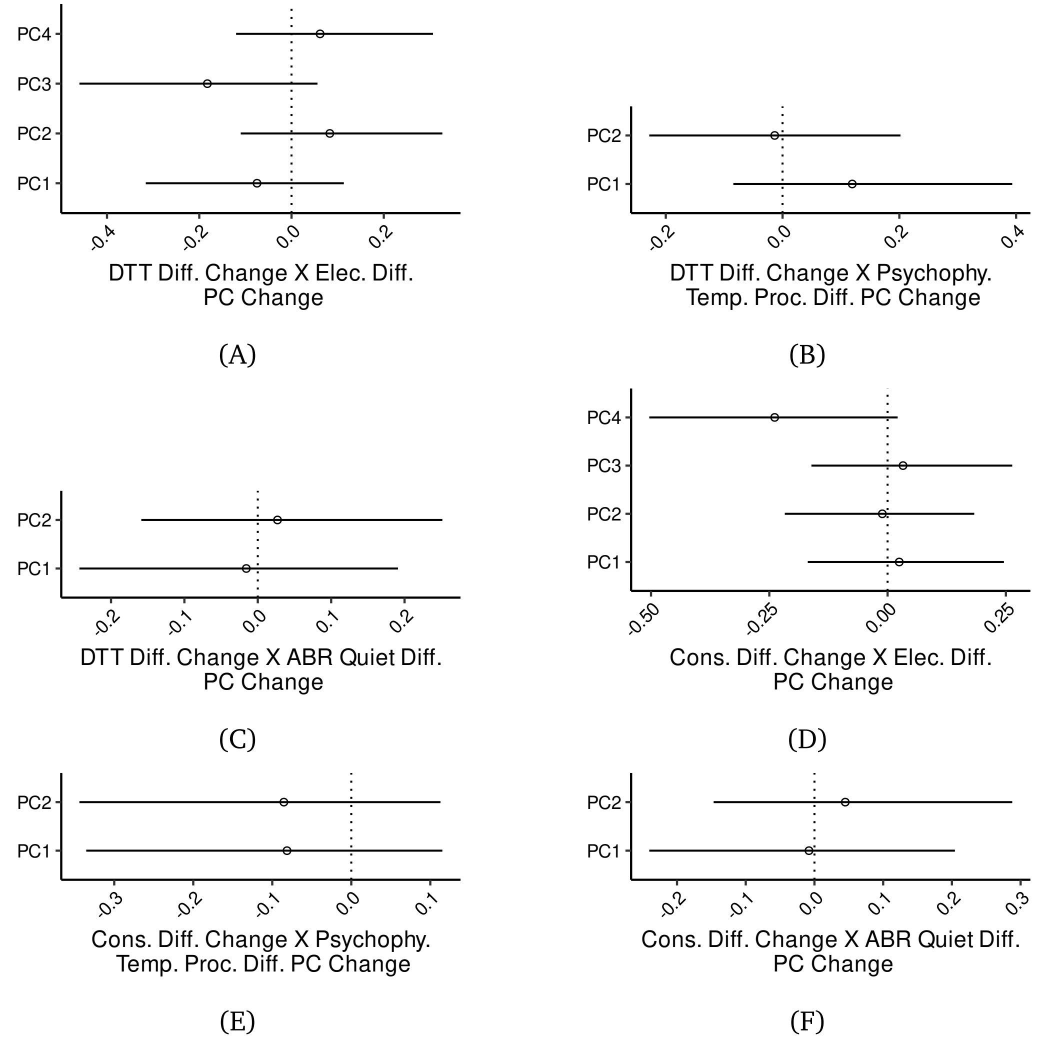

Figure S3: Posterior medians (circles) and 99% CIs for the effects of A) the electrophysiological difference measures (in HP masking noise) PCs on the DTT difference thresholds, B) the psychophysical temporal processing difference PCs on the DTT difference thresholds C) the ABR in quiet difference measures PCs on the DTT difference thresholds, D) the electrophysiological difference measures (in HP masking noise) PCs on the consonance difference measure, E) the psychophysical temporal processing difference PCs on the consonance difference measure F) the ABR in quiet difference measures PCs on the consonance difference measure.

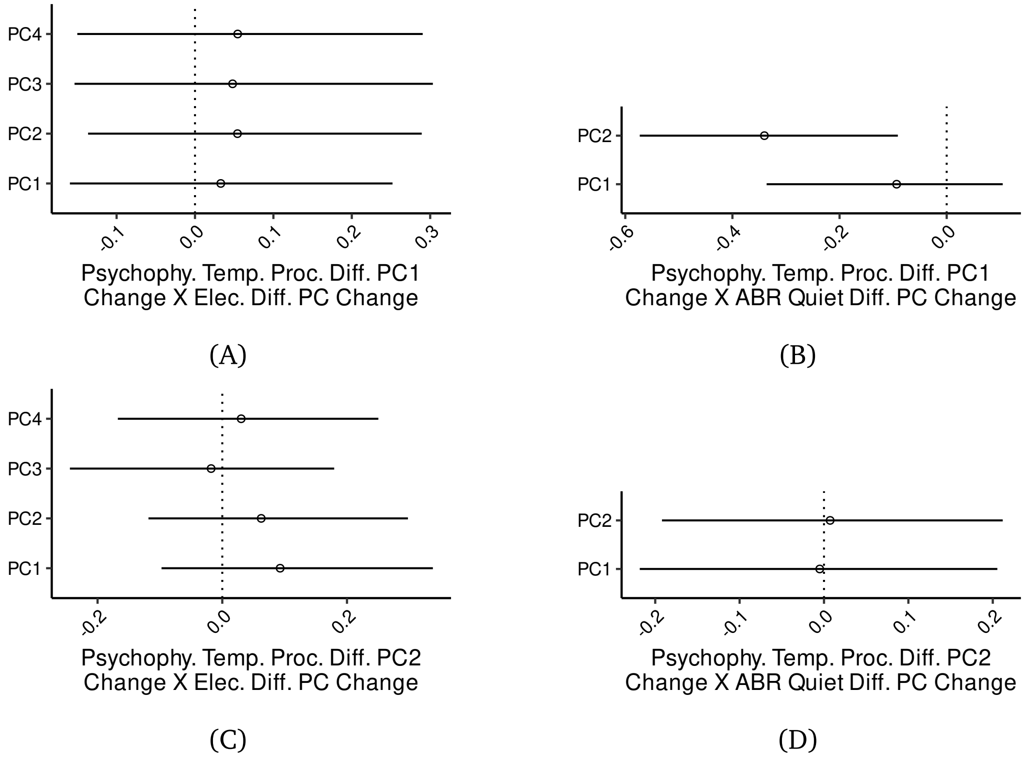

Figure S4: Posterior medians (circles) and 99% CIs for the effects of A) the electrophysiological difference measures (in HP masking noise) PCs on the pshychophysical difference measures PC1, B) the ABR in quiet difference measures PCs on the psychophysical difference measures PC1, C) the electrophysiological difference measures (in HP masking noise) PCs on the pshychophysical difference measures PC2, D) the ABR in quiet difference measures PCs on the psychophysical difference measures PC2.

2 Supplementary principal component analyses results

	Eigenvalue	% Variance	Cumulative % variance
PC1	2.87	47.82	47.82
PC2	1.12	18.73	66.55
PC3	0.78	13.02	79.56
PC4	0.59	9.90	89.46
PC5	0.42	6.96	96.43
PC6	0.21	3.57	100.00

Table S1: Eigenvalues, percentage of variance explained, and cumulative percentage of variance explained for the PCs extracted from the PCA of speech test scores.

	Eigenvalue	% Variance	Cumulative % variance
PC1	1.71	85.49	85.49
PC2	0.29	14.51	100.00

Table S2: Eigenvalues, percentage of variance explained, and cumulative percentage of variance explained for the PCs extracted from the PCA of consonance preference scores.

	Eigenvalue	% Variance	Cumulative % variance
PC1	6.99	38.81	38.81
PC2	2.18	12.13	50.94
PC3	1.54	8.56	59.49
PC4	1.15	6.39	65.89
PC5	1.02	5.66	71.55
PC6	0.85	4.73	76.28
PC7	0.70	3.89	80.17
PC8	0.55	3.08	83.25
PC9	0.50	2.79	86.04
PC10	0.46	2.56	88.60
PC11	0.42	2.33	90.93
PC12	0.36	2.00	92.93
PC13	0.35	1.95	94.89
PC14	0.26	1.46	96.35
PC15	0.22	1.21	97.56
PC16	0.19	1.06	98.61
PC17	0.15	0.82	99.43
PC18	0.10	0.57	100.00

Table S3: Eigenvalues, percentage of variance explained, and cumulative percentage of variance explained for the PCs extracted from the PCA of psychophysical temporal processing test scores.

	Eigenvalue	% Variance	Cumulative % variance
PC1	8.56	30.57	30.57
PC2	5.41	19.33	49.91
PC3	2.88	10.27	60.18
PC4	2.61	9.33	69.51
PC5	1.66	5.92	75.43
PC6	1.38	4.91	80.34
PC7	1.10	3.95	84.29
PC8	0.95	3.40	87.69
PC9	0.62	2.22	89.91
PC10	0.57	2.05	91.96
PC11	0.42	1.49	93.45
PC12	0.29	1.03	94.47
PC13	0.26	0.94	95.42
PC14	0.23	0.83	96.24
PC15	0.19	0.69	96.93
PC16	0.18	0.65	97.59
PC17	0.15	0.55	98.13
PC18	0.15	0.52	98.66
PC19	0.09	0.34	98.99
PC20	0.07	0.25	99.24
PC21	0.05	0.20	99.44
PC22	0.04	0.14	99.58
PC23	0.03	0.12	99.70
PC24	0.03	0.11	99.81
PC25	0.02	0.08	99.89
PC26	0.01	0.05	99.94
PC27	0.01	0.04	99.98
PC28	0.01	0.02	100.00

Table S4: Eigenvalues, percentage of variance explained, and cumulative percentage of variance explained for the PCs extracted from the PCA of electrophysiological measures.

2.1 Principal component analysis of cognitive measures

Table S5 shows the percentage of variance accounted for by each PC of the PCA on cognitive test scores. Table S6 shows the loadings (correlations) between each cognitive test and the four PCs extracted by the PCA. The first PC accounted for half of the variance, and was the only component retained on the basis of the results of parallel analysis. This component had high positive loadings on all the cognitive tests.

	Eigenvalue	% Variance	Cumulative % variance
PC1	2.00	50.00	50.00
PC2	0.96	23.97	73.97
PC3	0.62	15.41	89.37
PC4	0.43	10.63	100.00

Table S5: Eigenvalues, percentage of variance explained, and cumulative percentage of variance explained for the PCs extracted from the PCA of cognitive test scores.

	PC1	PC2	PC3	PC4
Dig. Span Forw.	0.69	-0.59	0.11	0.40
Dig. Span Backw.	0.79	-0.31	-0.27	-0.44
Raven Matrices	0.69	0.40	0.59	-0.11
Reading Span	0.64	0.60	-0.43	0.23

Table S6: Loadings (correlations) between PCs and variables of the PCA of cognitive tests scores.

2.2 Principal component analysis of auditory brainstem response in quiet measures

Table S7 shows the percentage of variance accounted for by each PC of the PCA on the ABR in quiet measures. Table S8 shows the loadings between each ABR in quiet measure and the first five PCs. The parallel analysis results indicated that three components, accounting together for 75% of the variance, could be reliably extracted. The first component accounted for 37% of the variance. This component had moderate or high positive loadings on all variables.

The second component accounted for 22% of the variance. This component had moderate positive loadings on the wave I measures, and negative loadings on the wave V measures (of moderate size, except for the earlobe montage at the low stimulus level, which had a small size).

The third component accounted for 15% of the variance. This component had positive loadings on the wave I and V measures at the low stimulus level (moderate for wave I and small for wave V), moderate negative loadings on the wave I measures at the high stimulus level, and small negative (ERL montage), or close to zero (TPR montage) loadings on the wave V measures at the high stimulus level.

	Eigenvalue	% Variance	Cumulative % variance
PC1	2.98	37.29	37.29
PC2	1.75	21.87	59.16
PC3	1.23	15.35	74.50
PC4	0.86	10.71	85.21
PC5	0.46	5.78	90.99
PC6	0.35	4.37	95.36
PC7	0.21	2.67	98.03
PC8	0.16	1.97	100.00

Table S7: Eigenvalues, percentage of variance explained, and cumulative percentage of variance explained for the PCs extracted from the PCA of ABR in quiet measures.

	PC1	PC2	PC3	PC4	PC5
ABRQ_HL_I_ERL	0.68	0.48	-0.46	0.08	0.01
ABRQ_HL_I_TPR	0.66	0.46	-0.52	0.04	-0.05
ABRQ_HL_V_ERL	0.66	-0.46	-0.22	-0.35	0.25
ABRQ_HL_V_TPR	0.72	-0.50	0.09	-0.31	0.01
ABRQ_LL_I_ERL	0.31	0.56	0.60	-0.25	0.35
ABRQ_LL_I_TPR	0.54	0.52	0.46	-0.07	-0.31
ABRQ_LL_V_ERL	0.54	-0.17	0.21	0.74	0.27
ABRQ_LL_V_TPR	0.68	-0.48	0.28	0.13	-0.33

Table S8: Loadings (correlations) between PCs and variables of the PCA of ABR in quiet measures. For brevity only the results for the first five PCs are shown.

2.3 Principal component analysis of psychophysical temporal processing difference measures

Table S9 shows the percentage of variance accounted for by each PC of the PCA on the psychophysical temporal processing difference measures. Table S10 shows the loadings between each difference measure and the first five PCs. The parallel analysis results indicated that two components, accounting together for 39% of the variance, could be reliably extracted. The first component accounted for 22% of the variance. This component had high positive loadings on the AMD tests, small/moderate positive loadings on the F0D, FD at 2 kHz and IPD MOD at 2 kHz tests, and close to zero loadings on the other tests (FD at 0.6 kHz, IPD MOD at 0.6 kHz, and IPD PT).

The second component, which accounted for 17% of the variance, had moderate/high positive loadings on the FD at 0.6 kHz, IPD MOD at 0.6 kHz, and IPD PT tests, small positive loadings on the IPD MOD at 2 kHz and AMD at 25 Hz tests, close to zero loadings on the F0D, FD at 2 kHz and AMD at 50 Hz tests, and a small negative loading on the AMD at 100 Hz test.

	Eigenvalue	% Variance	Cumulative % variance
PC1	1.98	22.05	22.05
PC2	1.53	17.02	39.06
PC3	1.17	12.96	52.02
PC4	1.01	11.24	63.26
PC5	0.96	10.62	73.88
PC6	0.85	9.44	83.32
PC7	0.57	6.37	89.69
PC8	0.51	5.71	95.40
PC9	0.41	4.60	100.00

Table S9: Eigenvalues, percentage of variance explained, and cumulative percentage of variance explained for the PCs extracted from the PCA of psychophysical temporal processing difference measures.

	PC1	PC2	PC3	PC4	PC5
AM_25Hz	0.75	0.15	-0.26	0.16	-0.07
AM_50Hz	0.80	0.07	-0.10	0.02	-0.16
AM_100Hz	0.73	-0.24	-0.26	-0.19	0.21
F0D	0.28	-0.08	0.67	0.51	-0.20
FD_PT_2kHz	0.25	0.05	0.49	-0.01	0.80
FD_PT_0.6kHz	0.01	0.56	-0.29	0.52	0.15
IPD_MOD_2kHz	0.33	0.13	0.47	-0.46	-0.35
IPD_MOD_0.6kHz	-0.04	0.73	-0.01	-0.45	0.14
IPD_PT	-0.01	0.75	0.16	0.09	-0.17

Table S10: Loadings (correlations) between PCs and variables of the PCA of psychophysical temporal processing difference measures. For brevity only the results for the first five PCs are shown.

2.4 Principal component analysis of electrophysiological difference measures

Table S11 shows the percentage of variance accounted for by each PC of the PCA on the electrophysiological difference measures. Table S12 shows the loadings between each difference measure and the first five PCs. The parallel analysis results indicated that four components, accounting together for 68% of the variance, could be reliably extracted. The first component accounted for 22% of the variance. This component had moderate/high positive loadings on most of the FFR measures, and loadings close to zero or small on the ABR measures.

The second component accounted for 20% of the variance. This component had moderate positive loadings on the FFR measures at 2 kHz and on the ABR wave V measures, moderate negative loadings on the FFR measures at 0.6 kHz, and small loadings on the ABR wave I measures.

The third component accounted for 14% of the variance. This component had high positive loadings on the ABR wave I measures, small positive loadings on two FFR measures (at 2 kHz with the mastoid montage, and at 0.6 kHz with the C7 montage), and loadings close to zero for all other measures.

The fourth component accounted for 12% of the variance. This component had moderate/high positive loadings for the wave V measures and low loadings for the wave I measures. The loadings of this component on the FFR measures were low/moderate, and tended to be negative for the measures at 2 kHz (with

the exception of the MST montage, which had a small positive loading), and positive for the measures at 0.6 kHz.

	Eigenvalue	% Variance	Cumulative % variance
PC1	2.67	22.27	22.27
PC2	2.34	19.52	41.78
PC3	1.66	13.84	55.62
PC4	1.49	12.42	68.04
PC5	0.99	8.24	76.29
PC6	0.90	7.53	83.82
PC7	0.58	4.85	88.67
PC8	0.49	4.09	92.76
PC9	0.34	2.85	95.61
PC10	0.27	2.22	97.83
PC11	0.17	1.38	99.21
PC12	0.09	0.79	100.00

Table S11: Eigenvalues, percentage of variance explained, and cumulative percentage of variance explained for the PCs extracted from the PCA of electrophysiological difference measures.

2.5 Principal component analysis of auditory brainstem response in quiet difference measures

Table S13 shows the percentage of variance accounted for by each PC of the PCA on the ABR in quiet difference measures. Table S14 shows the loadings between each difference measure and the first five PCs. The parallel analysis results indicated that two components, accounting together for 76% of the variance, could be reliably extracted. The first component accounted for 43% of the variance. This component had high positive loadings on the wave I measures and moderate positive loadings on the wave V measures.

The second component, which accounted for 33% of the variance, had high positive loadings on the wave V measures and small/moderate negative loadings on the wave I measures.

	PC1	PC2	PC3	PC4	PC5
ABR_I_ERL	-0.09	0.12	0.89	0.13	0.08
ABR_I_TPR	-0.15	-0.10	0.89	-0.07	0.07
ABR_V_ERL	0.03	0.48	-0.05	0.75	-0.20
ABR_V_TPR	-0.02	0.62	0.05	0.56	-0.24
FFR_ENV_2kHz_C7	0.38	0.45	0.05	-0.23	0.19
FFR_ENV_2kHz_ERL	0.59	0.56	0.03	-0.42	-0.04
FFR_ENV_2kHz_MST	0.40	0.44	-0.08	0.26	0.60
FFR_ENV_2kHz_TPR	0.68	0.46	0.16	-0.28	-0.14
FFR_ENV_0.6kHz_C7	0.44	-0.45	0.21	0.21	-0.29
FFR_ENV_0.6kHz_ERL	0.80	-0.45	0.03	0.16	-0.09
FFR_ENV_0.6kHz_MST	0.26	-0.44	-0.01	0.36	0.59
FFR_ENV_0.6kHz_TPR	0.79	-0.42	-0.02	0.13	-0.16

Table S12: Loadings (correlations) between PCs and variables of the PCA of electrophysiological difference measures. For brevity only the results for the first five PCs are shown.

	Eigenvalue	% Variance	Cumulative % variance
PC1	1.72	42.88	42.88
PC2	1.33	33.23	76.11
PC3	0.61	15.26	91.37
PC4	0.35	8.63	100.00

Table S13: Eigenvalues, percentage of variance explained, and cumulative percentage of variance explained for the PCs extracted from the PCA of ABR in quiet difference measures.

	PC1	PC2	PC3	PC4
ABRQ_I_ERL	0.78	-0.48	0.10	0.40
ABRQ_I_TPR	0.86	-0.29	-0.10	-0.41
ABRQ_V_ERL	0.42	0.71	0.56	-0.02
ABRQ_V_TPR	0.44	0.72	-0.53	0.12

Table S14: Loadings (correlations) between PCs and variables of the PCA of ABR in quiet difference measures. For brevity only the results for the first five PCs are shown.

3 Difference measures models

Dependent variable	Primary predictors
diff_CRM_CNT	diff_elec_PC1; diff_elec_PC2; diff_elec_PC3; diff_elec_PC4
diff_CRM_OFF	diff_elec_PC1; diff_elec_PC2; diff_elec_PC3; diff_elec_PC4
diff_DTT	diff_elec_PC1; diff_elec_PC2; diff_elec_PC3; diff_elec_PC4
diff_CRM_CNT	diff_psyphy_PC1; diff_psyphy_PC2
diff_CRM_OFF	diff_psyphy_PC1; diff_psyphy_PC2
diff_DTT	diff_psyphy_PC1; diff_psyphy_PC2
diff_CRM_CNT	diff_ABRQ_PC1; diff_ABRQ_PC2
diff_CRM_OFF	diff_ABRQ_PC1; diff_ABRQ_PC2
diff_DTT	diff_ABRQ_PC1; diff_ABRQ_PC2
diff_cons	diff_elec_PC1; diff_elec_PC2; diff_elec_PC3; diff_elec_PC4
diff_cons	diff_psyphy_PC1; diff_psyphy_PC2
diff_cons	diff_ABRQ_PC1; diff_ABRQ_PC2
diff_psyphy_PC1	diff_elec_PC1; diff_elec_PC2; diff_elec_PC3; diff_elec_PC4
diff_psyphy_PC2	diff_elec_PC1; diff_elec_PC2; diff_elec_PC3; diff_elec_PC4
diff_psyphy_PC1	diff_ABRQ_PC1; diff_ABRQ_PC2
diff_psyphy_PC2	diff_ABRQ_PC1; diff_ABRQ_PC2

Table S15: Synthetic overview of the MLR models used to assess relations between the difference measures. Each row refers to a different model. The first column indicates the dependent variable. The second column lists the main predictors of interest.

4 Correlations

Table S16 shows the correlations between the real-world hearing abilities variables, the psychophysical measures of temporal processing variables, and the electrophysiological measures of the study (excluding ABR latencies and ABR in quiet measures). For space reasons, the correlations with the electrophysiological measures are shown averaged across montages. As can be seen in tables S17, S18, and S19 the correlations between the different montages were generally high or moderate. Therefore correlations between the montage-specific electrophysiological measures and the other variables should not differ greatly from the correlations between the electrophysiological measures averaged across montages and the other variables that are shown in table S16. The correlations between the difference measures are shown in Table S20.

	CRM_HL_CNT	CRM_HL_OFF	CRM_LL_CNT	CRM_LL_OFF	DTT_HL	DTT_LL	AM_HL_100Hz	AM_HL_25Hz	AM_HL_50Hz	AM_LL_100Hz	AM_LL_25Hz	AM_LL_50Hz	F0D_HL	FD_HL_PT_2kHz	FD_HL_PT_0.6kHz	F0D_LL	FD_LL_PT_2kHz	FD_LL_PT_0.6kHz	IPD_HL_MOD_2kHz	IPD_HL_MOD_0.6kHz	IPD_HL_PT	IPD_LL_MOD_2kHz	IPD_LL_MOD_0.6kHz	IPD_LL_PT	cons_LL	cons_HL	ABR_HL_I	ABR_HL_V	ABR_LL_I	ABR_LL_V	FFR_ENV_MI0.7_2kHz	FFR_ENV_MI0.7_0.6kHz	FFR_ENV_MI1_2kHz	FFR_ENV_MI1_0.6kHz	FFR_TFS
CRM_HL_CNT	1.00	0.41	0.51	0.31	0.23	0.21	0.36	0.22	0.25	0.25	0.18	0.29	0.24	0.31	0.42	0.22	0.23	0.23	0.25	0.27	0.32	0.21	0.21	0.29	-0.22	-0.18	-0.15	-0.06	-0.09	-0.13	-0.13	0.01	-0.11	0.07	-0.09
CRM_HL_OFF	0.41	1.00	0.50	0.75	0.26	0.29	0.24	0.26	0.23	0.15	0.05	0.09	0.19	0.26	0.30	0.19	0.29	0.14	0.19	0.27	0.24	0.30	0.15	0.17	-0.08	-0.11	-0.00	0.04	-0.20	-0.10	-0.10	-0.17	-0.15	-0.12	-0.08
CRM_LL_CNT	0.51	0.50	1.00	0.57	0.10	0.23	0.31	0.28	0.23	0.28	0.12	0.21	0.20	0.35	0.35	0.40	0.43	0.19	0.21	0.14	0.18	0.26	0.28	0.18	-0.18	-0.23	0.11	-0.03	-0.17	-0.01	-0.16	-0.15	-0.17	-0.09	-0.12
CRM_LL_OFF	0.31	0.75	0.57	1.00	0.25	0.35	0.26	0.19	0.23	0.18	0.04	0.12	0.21	0.28	0.28	0.21	0.37	0.19	0.14	0.24	0.28	0.37	0.31	0.26	-0.15	-0.17	0.02	-0.05	-0.29	-0.07	-0.06	-0.26	-0.08	-0.19	-0.12
DTT_HL	0.23	0.26	0.10	0.25	1.00	0.41	0.24	0.17	0.17	-0.05	0.03	-0.04	0.18	0.15	0.20	0.00	0.15	0.27	0.11	0.16	0.10	0.10	0.22	0.18	-0.03	-0.03	-0.05	0.08	-0.03	-0.02	-0.01	-0.05	-0.00	0.03	0.04
DTT_LL	0.21	0.29	0.23	0.35	0.41	1.00	0.27	0.16	0.19	0.12	0.19	0.13	0.27	0.24	0.15	0.14	0.17	0.26	0.17	0.25	0.09	0.29	0.12	0.24	-0.23	-0.12	-0.05	-0.16	-0.34	0.01	-0.06	-0.10	-0.07	-0.08	-0.02
AM_HL_100Hz	0.36	0.24	0.31	0.26	0.24	0.27	1.00	0.66	0.79	0.67	0.52	0.63	0.46	0.46	0.38	0.22	0.27	0.56	0.39	0.31	0.37	0.25	0.36	0.41	-0.30	-0.41	0.14	0.04	-0.12	-0.12	0.02	-0.09	-0.00	-0.06	-0.21
AM_HL_25Hz	0.22	0.26	0.28	0.19	0.17	0.16	0.66	1.00	0.82	0.48	0.59	0.53	0.44	0.34	0.25	0.13	0.20	0.31	0.32	0.20	0.18	0.12	0.21	0.15	-0.15	-0.31	0.11	0.04	-0.10	-0.14	-0.04	-0.23	-0.04	-0.19	-0.05
AM_HL_50Hz	0.25	0.23	0.23	0.23	0.17	0.19	0.79	0.82	1.00	0.61	0.65	0.62	0.38	0.35	0.35	0.09	0.26	0.46	0.35	0.30	0.31	0.16	0.27	0.22	-0.23	-0.37	0.12	-0.02	-0.14	-0.11	-0.02	-0.19	-0.04	-0.17	-0.16
AM_LL_100Hz	0.25	0.15	0.28	0.18	-0.05	0.12	0.67	0.48	0.61	1.00	0.63	0.76	0.31	0.45	0.32	0.13	0.35	0.43	0.35	0.34	0.44	0.21	0.36	0.38	-0.27	-0.41	0.09	-0.02	-0.08	-0.06	0.07	-0.07	0.06	-0.03	-0.20
AM_LL_25Hz	0.18	0.05	0.12	0.04	0.03	0.19	0.52	0.59	0.65	0.63	1.00	0.68	0.29	0.30	0.15	0.10	0.19	0.39	0.28	0.23	0.30	0.15	0.24	0.27	-0.22	-0.33	0.04	-0.06	-0.04	-0.12	-0.06	-0.09	-0.04	-0.04	-0.14
AM_LL_50Hz	0.29	0.09	0.21	0.12	-0.04	0.13	0.63	0.53	0.62	0.76	0.68	1.00	0.19	0.32	0.15	0.08	0.28	0.38	0.35	0.30	0.42	0.22	0.27	0.36	-0.25	-0.24	0.04	-0.09	-0.00	-0.04	0.03	-0.05	0.04	-0.03	-0.18
F0D_HL	0.24	0.19	0.20	0.21	0.18	0.27	0.46	0.44	0.38	0.31	0.29	0.19	1.00	0.38	0.42	0.30	0.20	0.49	0.26	0.13	0.18	0.19	0.29	0.20	-0.16	-0.24	0.11	0.08	-0.09	-0.17	0.08	-0.17	0.06	-0.13	0.09
FD_HL_PT_2kHz	0.31	0.26	0.35	0.28	0.15	0.24	0.46	0.34	0.35	0.45	0.30	0.32	0.38	1.00	0.49	0.15	0.54	0.34	0.19	0.28	0.38	0.22	0.30	0.35	-0.09	-0.23	0.13	0.02	-0.06	0.02	0.17	0.07	0.18	0.07	-0.09
FD_HL_PT_0.6kHz	0.42	0.30	0.35	0.28	0.20	0.15	0.38	0.25	0.35	0.32	0.15	0.15	0.42	0.49	1.00	0.19	0.35	0.54	0.23	0.36	0.40	0.22	0.36	0.28	-0.10	-0.24	0.11	0.10	-0.09	-0.03	0.18	-0.04	0.19	0.01	0.00
F0D_LL	0.22	0.19	0.40	0.21	0.00	0.14	0.22	0.13	0.09	0.13	0.10	0.08	0.30	0.15	0.19	1.00	0.18	0.19	0.06	0.06	-0.00	0.12	0.01	0.08	-0.05	-0.02	-0.07	-0.13	-0.06	-0.17	-0.12	0.05	-0.13	0.09	0.04
FD_LL_PT_2kHz	0.23	0.29	0.43	0.37	0.15	0.17	0.27	0.20	0.26	0.35	0.19	0.28	0.20	0.54	0.35	0.18	1.00	0.24	0.21	0.19	0.34	0.31	0.27	0.32	-0.21	-0.27	0.02	-0.16	-0.22	-0.20	-0.07	-0.19	-0.08	-0.16	-0.15
FD_LL_PT_0.6kHz	0.23	0.14	0.19	0.19	0.27	0.26	0.56	0.31	0.46	0.43	0.39	0.38	0.49	0.34	0.54	0.19	0.24	1.00	0.22	0.28	0.36	0.15	0.44	0.37	-0.29	-0.31	0.17	0.10	-0.04	-0.06	0.05	-0.03	0.06	0.03	-0.10
IPD_HL_MOD_2kHz	0.25	0.19	0.21	0.14	0.11	0.17	0.39	0.32	0.35	0.35	0.28	0.35	0.26	0.19	0.23	0.06	0.21	0.22	1.00	0.45	0.38	0.67	0.25	0.40	-0.21	-0.29	0.07	-0.04	-0.20	-0.32	-0.04	-0.20	-0.08	-0.19	-0.16
IPD_HL_MOD_0.6kHz	0.27	0.27	0.14	0.24	0.16	0.25	0.31	0.20	0.30	0.34	0.23	0.30	0.13	0.28	0.36	0.06	0.19	0.28	0.45	1.00	0.55	0.53	0.40	0.44	-0.11	-0.14	-0.01	0.01	-0.15	-0.14	0.16	0.01	0.12	0.02	-0.19
IPD_HL_PT	0.32	0.24	0.18	0.28	0.10	0.09	0.37	0.18	0.31	0.44	0.30	0.42	0.18	0.38	0.40	-0.00	0.34	0.36	0.38	0.55	1.00	0.43	0.51	0.72	-0.25	-0.21	0.14	-0.02	-0.02	-0.08	0.05	-0.14	0.05	-0.13	-0.29
IPD_LL_MOD_2kHz	0.21	0.30	0.26	0.37	0.10	0.29	0.25	0.12	0.16	0.21	0.15	0.22	0.19	0.22	0.22	0.12	0.31	0.15	0.67	0.53	0.43	1.00	0.39	0.46	-0.22	-0.27	0.05	-0.03	-0.25	-0.32	-0.03	-0.13	-0.01	-0.15	-0.15
IPD_LL_MOD_0.6kHz	0.21	0.15	0.28	0.31	0.22	0.12	0.36	0.21	0.27	0.36	0.24	0.27	0.29	0.30	0.36	0.01	0.27	0.44	0.25	0.40	0.51	0.39	1.00	0.66	-0.22	-0.23	0.15	0.10	-0.10	0.14	0.00	-0.03	0.07	0.00	-0.01
IPD_LL_PT	0.29	0.17	0.18	0.26	0.18	0.24	0.41	0.15	0.22	0.38	0.27	0.36	0.20	0.35	0.28	0.08	0.32	0.37	0.40	0.44	0.72	0.46	0.66	1.00	-0.32	-0.23	0.08	-0.09	-0.20	-0.01	-0.05	-0.17	-0.02	-0.14	-0.21
cons_LL	-0.22	-0.08	-0.18	-0.15	-0.03	-0.23	-0.30	-0.15	-0.23	-0.27	-0.22	-0.25	-0.16	-0.09	-0.10	-0.05	-0.21	-0.29	-0.21	-0.11	-0.25	-0.22	-0.22	-0.32	1.00	0.71	-0.04	0.06	0.15	0.02	0.14	0.10	0.15	0.07	0.12
cons_HL	-0.18	-0.11	-0.23	-0.17	-0.03	-0.12	-0.41	-0.31	-0.37	-0.41	-0.33	-0.24	-0.24	-0.23	-0.24	-0.02	-0.27	-0.31	-0.29	-0.14	-0.21	-0.27	-0.23	-0.23	0.71	1.00	-0.07	-0.14	0.11	0.09	-0.03	0.14	-0.04	0.12	0.18
ABR_HL_I	-0.15	-0.00	0.11	0.02	-0.05	-0.05	0.14	0.11	0.12	0.09	0.04	0.04	0.11	0.13	0.11	-0.07	0.02	0.17	0.07	-0.01	0.14	0.05	0.15	0.08	-0.04	-0.07	1.00	0.46	0.20	0.20	0.15	-0.04	0.18	-0.04	0.03
ABR_HL_V	-0.06	0.04	-0.03	-0.05	0.08	-0.16	0.04	0.04	-0.02	-0.02	-0.06	-0.09	0.08	0.02	0.10	-0.13	-0.16	0.10	-0.04	0.01	-0.02	-0.03	0.10	-0.09	0.06	-0.14	0.46	1.00	0.36	0.13	0.23	0.12	0.20	0.15	0.07
ABR_LL_I	-0.09	-0.20	-0.17	-0.29	-0.03	-0.34	-0.12	-0.10	-0.14	-0.08	-0.04	-0.00	-0.09	-0.06	-0.09	-0.06	-0.22	-0.04	-0.20	-0.15	-0.02	-0.25	-0.10	-0.20	0.15	0.11	0.20	0.36	1.00	0.17	0.04	0.28	0.06	0.27	0.18
ABR_LL_V	-0.13	-0.10	-0.01	-0.07	-0.02	0.01	-0.12	-0.14	-0.11	-0.06	-0.12	-0.04	-0.17	0.02	-0.03	-0.17	-0.20	-0.06	-0.32	-0.14	-0.08	-0.32	0.14	-0.01	0.02	0.09	0.20	0.13	0.17	1.00	0.12	0.15	0.16	0.12	0.19
FFR_ENV_MI0.7_2kHz	-0.13	-0.10	-0.16	-0.06	-0.01	-0.06	0.02	-0.04	-0.02	0.07	-0.06	0.03	0.08	0.17	0.18	-0.12	-0.07	0.05	-0.04	0.16	0.05	-0.03	0.00	-0.05	0.14	-0.03	0.15	0.23	0.04	0.12	1.00	0.16	0.95	0.15	0.16
FFR_ENV_MI0.7_0.6kHz	0.01	-0.17	-0.15	-0.26	-0.05	-0.10	-0.09	-0.23	-0.19	-0.07	-0.09	-0.05	-0.17	0.07	-0.04	0.05	-0.19	-0.03	-0.20	0.01	-0.14	-0.13	-0.03	-0.17	0.10	0.14	-0.04	0.12	0.28	0.15	0.16	1.00	0.17	0.95	0.34
FFR_ENV_MI1_2kHz	-0.11	-0.15	-0.17	-0.08	-0.00	-0.07	-0.00	-0.04	-0.04	0.06	-0.04	0.04	0.06	0.18	0.19	-0.13	-0.08	0.06	-0.08	0.12	0.05	-0.01	0.07	-0.02	0.15	-0.04	0.18	0.20	0.06	0.16	0.95	0.17	1.00	0.18	0.18
FFR_ENV_MI1_0.6kHz	0.07	-0.12	-0.09	-0.19	0.03	-0.08	-0.06	-0.19	-0.17	-0.03	-0.04	-0.03	-0.13	0.07	0.01	0.09	-0.16	0.03	-0.19	0.02	-0.13	-0.15	0.00	-0.14	0.07	0.12	-0.04	0.15	0.27	0.12	0.15	0.95	0.18	1.00	0.35
FFR_TFS	-0.09	-0.08	-0.12	-0.12	0.04	-0.02	-0.21	-0.05	-0.16	-0.20	-0.14	-0.18	0.09	-0.09	0.00	0.04	-0.15	-0.10	-0.16	-0.19	-0.29	-0.15	-0.01	-0.21	0.12	0.18	0.03	0.07	0.18	0.19	0.16	0.34	0.18	0.35	1.00

Table S16: Correlations.

	ABR_HL_I_ERL	ABR_HL_I_TPR	ABR_HL_V_ERL	ABR_HL_V_TPR	ABR_LL_I_ERL	ABR_LL_I_TPR	ABR_LL_V_ERL	ABR_LL_V_TPR
ABR_HL_I_ERL	1.00	0.54	0.56	0.22	0.12	0.04	0.24	0.02
ABR_HL_I_TPR	0.54	1.00	0.31	0.18	0.30	0.27	0.24	0.19
ABR_HL_V_ERL	0.56	0.31	1.00	0.68	0.37	0.37	0.15	0.09
ABR_HL_V_TPR	0.22	0.18	0.68	1.00	0.23	0.17	0.13	0.08
ABR_LL_I_ERL	0.12	0.30	0.37	0.23	1.00	0.77	0.17	0.17
ABR_LL_I_TPR	0.04	0.27	0.37	0.17	0.77	1.00	0.11	0.15
ABR_LL_V_ERL	0.24	0.24	0.15	0.13	0.17	0.11	1.00	0.73
ABR_LL_V_TPR	0.02	0.19	0.09	0.08	0.17	0.15	0.73	1.00

Table S17: ABR correlations.

	FFR_ENV_MI0.7_2kHz_C7	FFR_ENV_MI0.7_2kHz_ERL	FFR_ENV_MI0.7_2kHz_MST	FFR_ENV_MI0.7_2kHz_TPR	FFR_ENV_MI0.7_0.6kHz_C7	FFR_ENV_MI0.7_0.6kHz_ERL	FFR_ENV_MI0.7_0.6kHz_MST	FFR_ENV_MI0.7_0.6kHz_TPR	FFR_ENV_MI1_2kHz_C7	FFR_ENV_MI1_2kHz_ERL	FFR_ENV_MI1_2kHz_MST	FFR_ENV_MI1_2kHz_TPR	FFR_ENV_MI1_0.6kHz_C7	FFR_ENV_MI1_0.6kHz_ERL	FFR_ENV_MI1_0.6kHz_MST	FFR_ENV_MI1_0.6kHz_TPR
FFR_ENV_MI0.7_2kHz_C7	1.00	0.70	0.58	0.64	0.23	0.21	0.12	0.20	0.89	0.68	0.57	0.67	0.20	0.18	0.09	0.19
FFR_ENV_MI0.7_2kHz_ERL	0.70	1.00	0.48	0.96	0.03	0.14	0.03	0.17	0.72	0.91	0.50	0.92	0.03	0.15	0.05	0.18
FFR_ENV_MI0.7_2kHz_MST	0.58	0.48	1.00	0.47	0.22	0.17	0.26	0.17	0.59	0.50	0.96	0.52	0.19	0.17	0.23	0.17
FFR_ENV_MI0.7_2kHz_TPR	0.64	0.96	0.47	1.00	-0.06	0.06	-0.03	0.10	0.66	0.86	0.49	0.94	-0.06	0.07	-0.03	0.11
FFR_ENV_MI0.7_0.6kHz_C7	0.23	0.03	0.22	-0.06	1.00	0.88	0.72	0.87	0.26	0.07	0.21	-0.02	0.94	0.85	0.73	0.83
FFR_ENV_MI0.7_0.6kHz_ERL	0.21	0.14	0.17	0.06	0.88	1.00	0.69	0.98	0.24	0.17	0.15	0.08	0.85	0.92	0.69	0.90
FFR_ENV_MI0.7_0.6kHz_MST	0.12	0.03	0.26	-0.03	0.72	0.69	1.00	0.68	0.17	0.06	0.22	-0.00	0.70	0.70	0.94	0.66
FFR_ENV_MI0.7_0.6kHz_TPR	0.20	0.17	0.17	0.10	0.87	0.98	0.68	1.00	0.26	0.18	0.16	0.12	0.85	0.92	0.69	0.93
FFR_ENV_MI1_2kHz_C7	0.89	0.72	0.59	0.66	0.26	0.24	0.17	0.26	1.00	0.78	0.63	0.75	0.24	0.23	0.12	0.24
FFR_ENV_MI1_2kHz_ERL	0.68	0.91	0.50	0.86	0.07	0.17	0.06	0.18	0.78	1.00	0.56	0.94	0.05	0.20	0.05	0.22
FFR_ENV_MI1_2kHz_MST	0.57	0.50	0.96	0.49	0.21	0.15	0.22	0.16	0.63	0.56	1.00	0.57	0.17	0.17	0.21	0.17
FFR_ENV_MI1_2kHz_TPR	0.67	0.92	0.52	0.94	-0.02	0.08	-0.00	0.12	0.75	0.94	0.57	1.00	-0.00	0.13	-0.01	0.17
FFR_ENV_MI1_0.6kHz_C7	0.20	0.03	0.19	-0.06	0.94	0.85	0.70	0.85	0.24	0.05	0.17	-0.00	1.00	0.90	0.74	0.86
FFR_ENV_MI1_0.6kHz_ERL	0.18	0.15	0.17	0.07	0.85	0.92	0.70	0.92	0.23	0.20	0.17	0.13	0.90	1.00	0.74	0.97
FFR_ENV_MI1_0.6kHz_MST	0.09	0.05	0.23	-0.03	0.73	0.69	0.94	0.69	0.12	0.05	0.21	-0.01	0.74	0.74	1.00	0.71
FFR_ENV_MI1_0.6kHz_TPR	0.19	0.18	0.17	0.11	0.83	0.90	0.66	0.93	0.24	0.22	0.17	0.17	0.86	0.97	0.71	1.00

Table S18: FFR ENV correlations.

	FFR_TFS_C7	FFR_TFS_ERL	FFR_TFS_MST	FFR_TFS_TPR
FFR_TFS_C7	1.00	0.77	0.75	0.74
FFR_TFS_ERL	0.77	1.00	0.84	0.95
FFR_TFS_MST	0.75	0.84	1.00	0.81
FFR_TFS_TPR	0.74	0.95	0.81	1.00

Table S19: FFR TFS correlations.

	CRM_CNT	CRM_OFF	DTT	AM_25Hz	AM_50Hz	AM_100Hz	F0D	FD_PT_2kHz	FD_PT_0.6kHz	IPD_MOD_2kHz	IPD_MOD_0.6kHz	IPD_PT	cons	ABR_I_ERL	ABR_I_TPR	ABR_V_ERL	ABR_V_TPR	ABRQ_I_ERL	ABRQ_I_TPR	ABRQ_V_ERL	ABRQ_V_TPR	FFR_ENV_2kHz_C7	FFR_ENV_2kHz_ERL	FFR_ENV_2kHz_MST	FFR_ENV_2kHz_TPR	FFR_ENV_0.6kHz_C7	FFR_ENV_0.6kHz_ERL	FFR_ENV_0.6kHz_MST	FFR_ENV_0.6kHz_TPR
CRM_CNT	1.00	0.22	0.11	-0.08	0.01	0.14	0.13	0.15	0.09	0.11	0.14	0.00	0.11	-0.16	-0.20	0.06	0.03	-0.07	0.09	-0.02	-0.02	-0.18	-0.01	-0.05	-0.07	-0.06	-0.03	-0.08	-0.01
CRM_OFF	0.22	1.00	0.07	0.17	0.09	0.05	-0.01	0.06	0.14	0.15	0.24	0.14	-0.05	-0.00	-0.13	0.16	0.11	0.12	0.05	-0.03	-0.11	0.06	0.18	0.16	0.17	0.06	0.09	-0.01	0.10
DTT	0.11	0.07	1.00	0.15	0.10	0.13	0.07	-0.05	0.04	0.15	-0.15	0.11	-0.13	-0.21	-0.25	0.23	0.12	-0.09	0.01	0.01	0.03	-0.09	-0.02	0.01	-0.04	-0.17	-0.19	0.04	-0.19
AM_25Hz	-0.08	0.17	0.15	1.00	0.45	0.40	0.09	0.05	0.17	0.14	0.01	0.03	-0.11	0.14	0.08	0.19	0.00	0.03	-0.01	-0.19	-0.17	-0.02	0.05	0.11	0.08	-0.14	0.01	0.19	0.03
AM_50Hz	0.01	0.09	0.10	0.45	1.00	0.46	0.14	0.06	0.03	0.19	-0.02	0.08	-0.24	0.23	0.22	0.11	0.11	-0.07	-0.15	-0.31	-0.32	0.06	0.08	0.06	0.15	-0.07	-0.06	0.08	-0.07
AM_100Hz	0.14	0.05	0.13	0.40	0.46	1.00	0.02	0.13	-0.13	0.04	-0.05	-0.16	0.00	0.13	0.08	0.16	0.03	0.01	0.03	-0.18	-0.32	-0.03	0.03	0.08	0.03	-0.08	0.01	0.05	0.06
F0D	0.13	-0.01	0.07	0.09	0.14	0.02	1.00	0.14	-0.05	0.12	-0.15	0.07	-0.10	0.09	0.08	0.09	0.11	0.05	-0.07	-0.30	-0.11	-0.05	0.03	0.01	-0.03	-0.02	0.02	0.15	-0.01
FD_PT_2kHz	0.15	0.06	-0.05	0.05	0.06	0.13	0.14	1.00	0.01	0.09	0.05	0.00	-0.09	-0.09	-0.07	0.11	-0.14	0.04	-0.02	-0.15	-0.14	-0.08	-0.12	0.01	-0.11	0.16	0.10	0.03	0.10
FD_PT_0.6kHz	0.09	0.14	0.04	0.17	0.03	-0.13	-0.05	0.01	1.00	-0.07	0.15	0.17	-0.16	0.03	-0.04	0.04	-0.11	0.06	0.06	-0.03	0.08	-0.03	0.02	-0.06	-0.02	0.09	0.04	0.02	-0.02
IPD_MOD_2kHz	0.11	0.15	0.15	0.14	0.19	0.04	0.12	0.09	-0.07	1.00	0.11	0.01	-0.06	-0.05	0.02	0.03	-0.03	0.06	-0.02	-0.25	-0.09	0.14	0.20	0.14	0.20	-0.24	-0.09	-0.08	0.00
IPD_MOD_0.6kHz	0.14	0.24	-0.15	0.01	-0.02	-0.05	-0.15	0.05	0.15	0.11	1.00	0.35	-0.02	-0.02	-0.18	0.04	0.14	0.12	0.12	0.01	0.02	0.25	0.14	0.31	0.16	0.05	0.06	0.06	0.15
IPD_PT	0.00	0.14	0.11	0.03	0.08	-0.16	0.07	0.00	0.17	0.01	0.35	1.00	-0.12	-0.16	-0.18	0.13	0.13	-0.16	-0.05	0.03	-0.03	0.00	0.01	0.25	0.12	-0.02	-0.01	0.19	-0.01
cons	0.11	-0.05	-0.13	-0.11	-0.24	0.00	-0.10	-0.09	-0.16	-0.06	-0.02	-0.12	1.00	0.03	-0.01	-0.30	-0.19	-0.09	0.04	0.09	0.03	0.06	0.08	0.04	0.11	0.01	-0.03	-0.08	0.01
ABR_I_ERL	-0.16	-0.00	-0.21	0.14	0.23	0.13	0.09	-0.09	0.03	-0.05	-0.02	-0.16	0.03	1.00	0.63	0.11	0.09	0.10	-0.03	-0.08	-0.08	0.03	-0.01	0.04	0.05	0.09	-0.09	-0.04	-0.09
ABR_I_TPR	-0.20	-0.13	-0.25	0.08	0.22	0.08	0.08	-0.07	-0.04	0.02	-0.18	-0.18	-0.01	0.63	1.00	-0.16	-0.01	-0.08	-0.25	-0.15	-0.13	-0.07	-0.09	-0.15	0.02	0.04	-0.02	0.02	-0.09
ABR_V_ERL	0.06	0.16	0.23	0.19	0.11	0.16	0.09	0.11	0.04	0.03	0.04	0.13	-0.30	0.11	-0.16	1.00	0.60	0.13	0.02	-0.08	-0.08	-0.03	0.01	0.27	0.09	-0.04	-0.07	-0.04	-0.04
ABR_V_TPR	0.03	0.11	0.12	0.00	0.11	0.03	0.11	-0.14	-0.11	-0.03	0.14	0.13	-0.19	0.09	-0.01	0.60	1.00	-0.11	-0.21	-0.08	-0.19	0.15	0.10	0.21	0.14	-0.11	-0.16	-0.14	-0.16
ABRQ_I_ERL	-0.07	0.12	-0.09	0.03	-0.07	0.01	0.05	0.04	0.06	0.06	0.12	-0.16	-0.09	0.10	-0.08	0.13	-0.11	1.00	0.63	0.04	-0.00	0.02	-0.08	-0.07	-0.09	0.12	0.06	0.03	0.10
ABRQ_I_TPR	0.09	0.05	0.01	-0.01	-0.15	0.03	-0.07	-0.02	0.06	-0.02	0.12	-0.05	0.04	-0.03	-0.25	0.02	-0.21	0.63	1.00	0.12	0.17	0.02	-0.09	-0.08	-0.10	0.22	0.12	0.01	0.19
ABRQ_V_ERL	-0.02	-0.03	0.01	-0.19	-0.31	-0.18	-0.30	-0.15	-0.03	-0.25	0.01	0.03	0.09	-0.08	-0.15	-0.08	-0.08	0.04	0.12	1.00	0.40	-0.15	-0.10	-0.04	-0.10	0.14	0.11	0.06	0.12
ABRQ_V_TPR	-0.02	-0.11	0.03	-0.17	-0.32	-0.32	-0.11	-0.14	0.08	-0.09	0.02	-0.03	0.03	-0.08	-0.13	-0.08	-0.19	-0.00	0.17	0.40	1.00	-0.01	-0.10	-0.16	-0.14	0.11	-0.08	-0.07	-0.03
FFR_ENV_2kHz_C7	-0.18	0.06	-0.09	-0.02	0.06	-0.03	-0.05	-0.08	-0.03	0.14	0.25	0.00	0.06	0.03	-0.07	-0.03	0.15	0.02	0.02	-0.15	-0.01	1.00	0.38	0.34	0.34	0.11	0.04	-0.13	0.00
FFR_ENV_2kHz_ERL	-0.01	0.18	-0.02	0.05	0.08	0.03	0.03	-0.12	0.02	0.20	0.14	0.01	0.08	-0.01	-0.09	0.01	0.10	-0.08	-0.09	-0.10	-0.10	0.38	1.00	0.30	0.81	-0.13	0.16	-0.18	0.17
FFR_ENV_2kHz_MST	-0.05	0.16	0.01	0.11	0.06	0.08	0.01	0.01	-0.06	0.14	0.31	0.25	0.04	0.04	-0.15	0.27	0.21	-0.07	-0.08	-0.04	-0.16	0.34	0.30	1.00	0.25	-0.09	0.13	0.15	0.09
FFR_ENV_2kHz_TPR	-0.07	0.17	-0.04	0.08	0.15	0.03	-0.03	-0.11	-0.02	0.20	0.16	0.12	0.11	0.05	0.02	0.09	0.14	-0.09	-0.10	-0.10	-0.14	0.34	0.81	0.25	1.00	0.07	0.24	-0.08	0.29
FFR_ENV_0.6kHz_C7	-0.06	0.06	-0.17	-0.14	-0.07	-0.08	-0.02	0.16	0.09	-0.24	0.05	-0.02	0.01	0.09	0.04	-0.04	-0.11	0.12	0.22	0.14	0.11	0.11	-0.13	-0.09	0.07	1.00	0.49	0.20	0.43
FFR_ENV_0.6kHz_ERL	-0.03	0.09	-0.19	0.01	-0.06	0.01	0.02	0.10	0.04	-0.09	0.06	-0.01	-0.03	-0.09	-0.02	-0.07	-0.16	0.06	0.12	0.11	-0.08	0.04	0.16	0.13	0.24	0.49	1.00	0.33	0.88
FFR_ENV_0.6kHz_MST	-0.08	-0.01	0.04	0.19	0.08	0.05	0.15	0.03	0.02	-0.08	0.06	0.19	-0.08	-0.04	0.02	-0.04	-0.14	0.03	0.01	0.06	-0.07	-0.13	-0.18	0.15	-0.08	0.20	0.33	1.00	0.28
FFR_ENV_0.6kHz_TPR	-0.01	0.10	-0.19	0.03	-0.07	0.06	-0.01	0.10	-0.02	0.00	0.15	-0.01	0.01	-0.09	-0.09	-0.04	-0.16	0.10	0.19	0.12	-0.03	0.00	0.17	0.09	0.29	0.43	0.88	0.28	1.00

Table S20: Correlations between the difference measures.
